# Particle filters for high‐dimensional geoscience applications: A review

**DOI:** 10.1002/qj.3551

**Published:** 2019-05-21

**Authors:** Peter Jan van Leeuwen, Hans R. Künsch, Lars Nerger, Roland Potthast, Sebastian Reich

**Affiliations:** ^1^ Department of Meteorology and National Centre for Earth Observation University of Reading Reading UK; ^2^ Department of Atmospheric Sciences Colorado State University Fort Collins Colorado USA; ^3^ Seminar für Statistik ETH Zurich Swiszerland; ^4^ Alfred Wegener Institute, Helmholtz Center for Polar and Marine Research Bremerhaven Germany; ^5^ Deutscher Wetterdienst Offenbach Germany; ^6^ Institut für Mathematik Universit'´at Potsdam Potsdam Germany

**Keywords:** hybrids, localization, nonlinear data assimilation, particle filters, proposal densities

## Abstract

Particle filters contain the promise of fully nonlinear data assimilation. They have been applied in numerous science areas, including the geosciences, but their application to high‐dimensional geoscience systems has been limited due to their inefficiency in high‐dimensional systems in standard settings. However, huge progress has been made, and this limitation is disappearing fast due to recent developments in proposal densities, the use of ideas from (optimal) transportation, the use of localization and intelligent adaptive resampling strategies. Furthermore, powerful hybrids between particle filters and ensemble Kalman filters and variational methods have been developed. We present a state‐of‐the‐art discussion of present efforts of developing particle filters for high‐dimensional nonlinear geoscience state‐estimation problems, with an emphasis on atmospheric and oceanic applications, including many new ideas, derivations and unifications, highlighting hidden connections, including pseudo‐code, and generating a valuable tool and guide for the community. Initial experiments show that particle filters can be competitive with present‐day methods for numerical weather prediction, suggesting that they will become mainstream soon.

## 1 INTRODUCTION

Data assimilation for geoscience applications, such as weather or ocean prediction, is a slowly maturing field. Even the linear data assimilation problem cannot be solved adequately because of the size of the problem. Typically, global‐scale numerical weather prediction needs estimation of over 10^9^ state variables, assimilating over 10^7^ observations every 6–12 hr. Existing methods like 4DVar do not provide accurate uncertainty estimates and need efficient pre‐conditioners, while Ensemble Kalman Filters (EnKFs) heavily rely on somewhat *adhoc* fixes like localization and inflation to find accurate estimates. Hybrids of variational and ensemble Kalman filter methods are a step forward, although localization and inflation are still needed in realistic applications. An extra complication is localization over time needed in ensemble smoothers like the Ensemble Kalman Smoother and four‐dimensional ensemble‐variational data assimilation system (4DEnsVar) when the fluid flow is strong: what is local at observation time is not necessary local at the start of the assimilation window because the observation influence is advected with the flow. Furthermore, the recent surge of papers on accurate treatment of observation errors shows that a long way is still ahead of us to solve even the (close to) linear data assimilation problem.

Although these problems are formidable, another difficulty arises from the fact that the problem is typically nonlinear, and, with increasing model resolution and more complex observation operators, increasingly so. Both variational and Kalman‐filter‐like methods have difficulty handling nonlinear problems. Variational methods can easily fail when the cost function is multimodal, and are hampered by the assumption that the prior probability density function (pdf) of the state is assumed to be Gaussian. EnKFs make the explicit assumption that the prior pdf and the likelihood of the observations as function of the state are Gaussian, or, somewhat equivalently, assume that the analysis is a linear combination of prior state and observations. Both methods have been shown to fail for nonlinear data assimilation problems in low‐dimensional systems, and both have been reported to have serious difficulties in numerical weather prediction at the convective scale where the model resolution is only a few km. Particle filters hold the promise of fully nonlinear data assimilation without any assumption on prior or likelihood, and recent textbooks like Reich and Cotter (2015), Nakamura and Potthast (2015), and van Leeuwen *et al.* (2015) provide useful introductions to data assimilation in general, and particle filters in particular.

Other fully nonlinear data assimilation methods are Markov chain Monte‐Carlo (MCMC) methods that draw directly from the posterior in a sequential way, so one sample after the other, after a burn‐in period; e.g. Robert and Cassela (2004) or van Leeuwen *et al.* (2015) give a geophysics‐friendly introduction. The samples are correlated, often 100*%* when the new sample is not accepted, making them very inefficient in high‐dimensional systems. This is why we concentrate on particle filtering here.

The standard or bootstrap particle filter can be described as follows. The starting point is an ensemble of size *N* of model states $x_{i}^{n}\in {ℜ}^{N_{x}}$, called particles, that represent the prior probability density function (pdf) *p*(**x** ^*n*^), as

(1) $$p(x^{n})\approx \overset{N}{\underset{i=1}{\sum }}\frac{1}{N}\delta (x^{n}-x_{i}^{n}).$$

Between observations, each of these particles is propagated forward from time *n* − 1 to time *n* with the typically nonlinear model equations

(2) $$x^{n}=f(x^{n-1})+\beta^{n}$$

in which *f*(..) denotes the deterministic model, and ***β*** ^*n*^ is a random forcing representing missing physics, discretization errors, etc. In this paper we assume this model noise to be additive, but one could also consider multiplicative noise in which ***β*** ^*n*^ is a function of the state of the system. We assume that the pdf from which the ***β*** ^*n*^ are drawn is known; typically a Gaussian *N*(0,**Q**).

At observation times the true system is observed via:

(3) $$y^{n}=H(x_{\text{true}}^{n})+\epsilon^{n},$$

in which the observation errors ***ϵ*** ^*n*^ are random vectors representing measurement errors and possibly representation errors. Again we assume that these errors have known characteristics, often Gaussian, so for example, ***ϵ*** ^*n*^∼*N*(0,**R**). These observations $y^{n}\in {ℜ}^{N_{y}}$ are assimilated by multiplying the prior pdf above with the likelihood of each possible state, that is, the probability density *p*(**y** ^*n*^|**x** ^*n*^) of the observation vector given each possible model state, following Bayes' theorem:

(4) $$p(x^{n}|y^{n})=\frac{p(y^{n}|x^{n})}{p(y^{n})}p(x^{n}),$$

in which *p*(**x** ^*n*^|**y** ^*n*^) is the posterior pdf, the holy grail of data assimilation. To avoid confusion, it is good to realise that the true state is not a random variable when we apply Bayes' theorem. It is a realization of a process, which could be random or deterministic, from which we then take noisy observations. Instead, Bayes' theorem is a statement of what we think the true state might be. Since the pdf of the ***ϵ*** ^*n*^ is known and Bayes' theorem is a statement for each possible state **x** ^*n*^ to be the true state, *p*(**y** ^*n*^|**x** ^*n*^) is the pdf of **y** ^*n*^ given that the true state vector would be **x** ^*n*^. In general, since for a given state **x** ^*n*^ the observation **y** ^*n*^ is equal to the observation error ***ϵ*** shifted by **H**(**x** ^*n*^), we find (e.g. van Leeuwen 2015):

(5) $$p(y^{n}|x^{n})=p_{\epsilon }\{y^{n}-H(x^{n})\}.$$

If we insert our particle representation of the prior into this theorem we find:

(6) $$p(x^{n}|y^{n})\approx \overset{N}{\underset{i=1}{\sum }}w_{i}\delta (x^{n}-x_{i}^{n}),$$

in which the particle weights *w*_*i*_ are given by:

(7) $$w_{i}^{n}=\frac{p(y^{n}|x_{i}^{n})}{Np(y^{n})}=\frac{p(y^{n}|x_{i}^{n})}{N\int p(y^{n}|x^{n})p(x^{n})\;dx^{n}}\approx \frac{p(y^{n}|x_{i}^{n})}{\underset{j}{\sum }p(y^{n}|x_{j}^{n})}.$$

**Figure 1:**
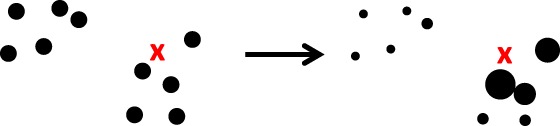
The standard particle filter. Left: the prior particles (dots), with one observation, denoted by the red cross. Right: the posterior particles, the larger the dot the larger its weight. Note that the particles do not move in state space, they are just reweighted [Colour figure can be viewed at wileyonlinelibrary.com]

Since all terms are known explicitly, we can just calculate this as a number. The self‐normalization in the last part of Equation 7 is consistent with the notion that, for a proper representation of a pdf, the sum of the weights should be equal to one, so that the integral over the whole state space of the particle representation of the pdf is equal to one. Figure 1 depicts the working of this filter.

Propagating the particles $x_{i}^{n}$ to the next observation time *n* + 1 gives a weighted representation of the prior at time *n* + 1. Assimilating the observation at time *n* + 1 by Bayes' theorem leads to a modification of the weights (e.g. Doucet *et al.* 2001 or van Leeuwen 2009):

(8) $$w_{i}^{n+1}=w_{i}^{n}\frac{p(y^{n+1}|x_{i}^{n+1})}{\underset{j}{\sum }p(y^{n+1}|x_{j}^{n+1})}.$$

Even in low‐dimensional applications, the variation of the weights increases with the number of assimilation steps. Eventually one particle has a much higher weight than all the others. To prevent this, resampling can be used before propagation to obtain equally weighted particles. This duplicates high‐weight particles and abandons low‐weight particles. After resampling, some of the particles have identical values, but if the model contains a stochastic component and independent random forcings are used for different particles, diversity is restored; e.g. Doucet *et al.* (2001) or van Leeuwen (2009) give details. Algorithm 1 illustrates the steps.


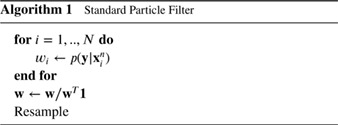


A simple resampling scheme using only one draw from a uniform distribution *U* is presented in Algorithm 2.


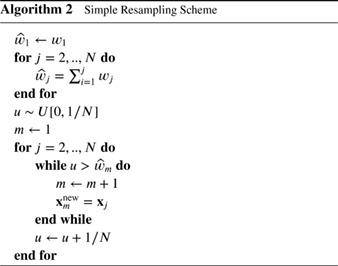


In high‐dimensional problems, the weights vary enormously even at one observation time, and typically one particle obtains a much higher weight than all the others. Snyder *et al.*, 2008 (2008, 2015) have shown that the number of particles needed to avoid a weight collapse, in which one particle gets weight 1 and the rest of the weights very close to zero, has to grow exponentially with the dimension of the observations **y** for a large class of particle filters. If the weights collapse, all particles are identical after resampling, and all diversity is lost. From this discussion it becomes clear that, for particle filters to work, we need to ensure that their weights remain similar.

In this review we will discuss four basic ways to make progress on this fundamental problem of weight degeneracy. In the first one, we explore the so‐called proposal‐density freedom to steer particles through state space such that they obtain very similar weights, e.g. Doucet *et al.* (2001). As pointed out by e.g. Snyder *et al.* (2008), there are fundamental problems when applying these techniques to the high‐dimensional geoscience applications. We will examine the issue in detail and discuss so‐called equal‐weight particle filters, which point towards new ways to formulate and attack the degeneracy problem.

The second approach transforms the prior particles into particles from the posterior, either in one go, or via a more smooth transformation process (Reich, 2013). While the one‐step approaches can be shown to fail in high‐dimensional settings, they do lend themselves very naturally to localization. The more smooth multi‐step transition variants seem to be able to avoid the degeneracy problem without localization, and are an interesting new development.

The third, more straightforward from the geoscience experience, approach is to introduce localization in particle filters. While initial implementations were discouraging (e.g. van Leeuwen 2009), new formulations have shown remarkable successes, such that localized particle filters are now tested in global operational numerical weather prediction systems (e.g. Potthast *et al.* 2019).

The fourth approach is to abandon the idea of using pure particle filters and combine them with EnKFs. This should not be confused with using EnKFs in proposal densities. Several variants exist, such as second‐order exact filters, in which only the first two moments are estimated, sequential versions in which first an EnKF is used and the posterior EnKF ensemble is used as input for the particle filter, or vice versa, and combinations in which localized weights are calculated and, dependent on the effective ensemble size, a full particle filter, an EnKF, or a combination of both is used.

These four variants form the basis of the following four sections. Each section contains a critical discussion of the approximations and remaining major issues. It should be noted that the pseudo‐code provided does not give the most efficient implementation of the different particle filters, but is rather an illustration of the computational steps involved. Efficient pseudo‐code for some of the more complex schemes can be found in Vetra‐Carvalho *et al.* (2018). The paper is closed with a concluding section and an outlook of what possible next steps could be.

## 2 PROPOSAL DENSITY PARTICLE FILTERS

Ideally we draw independent samples directly from the posterior pdf because the samples would all have equal weight automatically. This can only be done, however, when the shape of the posterior pdf is known and when it is easy to draw from the posterior. An example of this is a Gaussian prior combined with a linear Gaussian likelihood. Under these assumptions the posterior is also Gaussian and the mean and covariance can be calculated directly from the prior using the Kalman update equations. EnKFs make use of this result and draw directly from that pdf, which is why all posterior particles have equal weights in an EnKF.

The standard particle filter draws particles from the prior. These then have to be modified to become particles of the posterior via the weighting with the likelihood. This is a general procedure in statistics called importance sampling: one draws from an approximation of the pdf one is interested in, and corrects for this via so‐called importance weights.

In the introduction we argued that drawing from the prior leads to weights that vary too much: typically, in high‐dimensional problems with numerous independent observations one particle gets weight 1, and all other particles have a weight very close to zero. However, we could explore the idea of importance sampling on the transition from one time to the next. When the numerical model is not deterministic but stochastic we have the freedom to change the model equations to move the particles to those parts of state space where we want them to be, for instance closer to the observations.

Mathematically this works as follows. Assume we have observations at time *n*, so Bayes' theorem at time *n* is given by Equation 4. If the model is stochastic, we can write the prior as

(9) $$p(x^{n})=\int p(x^{n}|x^{n-1})p(x^{n-1})\;dx^{n-1},$$

where *p*(**x** ^*n*^|**x** ^*n* − 1^) is the transition density, the pdf of the state at time *n* when the state at time *n* − 1 is known. For instance, if the model error is additive and the model equation is given by Equation 2, it holds that

(10) $$p(x^{n}|x^{n-1})=p_{\beta }\left\{x^{n}-f(x^{n-1})\right\}.$$

Often the model errors are assumed to be Gaussian ***β***∼*N*(0,**Q**), and we find

(11) $$p(x^{n}|x^{n-1})=N\{f(x^{n-1}),Q\},$$

but the method is more general than that.

Assume now that at time *n* − 1 we have a set of weighted particles as in Equation 1, but with weights $w_{i}^{n-1}$ instead of 1/*N*. We can evaluate the expression Equation 9 for the prior as a weighted mixture of transition densities

(12) $$p(x^{n})\approx \overset{N}{\underset{i=1}{\sum }}w_{i}^{n-1}p(x^{n}|x_{i}^{n-1}).$$

In the following we neglect the approximation error at time *n* − 1 and assume that Equation 12 is exact. This is not necessarily a good approximation, especially when the number of particles is small. On the other hand, it is consistent with the particle filter approximation in the first place, and one of the few things one can do. By Bayes' formula 4, the posterior can then be written as:

(13) $$p(x^{n}|y^{n})\approx \overset{N}{\underset{i=1}{\sum }}w_{i}^{n-1}\frac{p(y^{n}|x^{n})}{p(y^{n})}p(x^{n}|x_{i}^{n-1}).$$

In the standard particle filter, one makes one draw from $p(x^{n}|x_{i}^{n-1})$ for each *i*, and we know that this leads to ensemble collapse for high‐dimensional systems. However, now the prior particles at time *n* are allowed to arise from following a different model equation. This works as follows. We can multiply and divide Equations 12 and 13 by a so‐called proposal density *q*(**x** ^*n*^|**x** ^*n* − 1^,**y** ^*n*^), leading to:

(14) $$p(x^{n})\approx \overset{N}{\underset{i=1}{\sum }}w_{i}^{n-1}\frac{p(x^{n}|x_{i}^{n-1})}{q(x^{n}|x_{i}^{n-1},y^{n})}q(x^{n}|x_{i}^{n-1},y^{n})$$

and

(15) $$p(x^{n}|y^{n})\approx \overset{N}{\underset{i=1}{\sum }}w_{i}^{n-1}\frac{p(y^{n}|x^{n})}{p(y^{n})}\frac{p(x^{n}|x_{i}^{n-1})}{q(x^{n}|x_{i}^{n-1},y^{n})}q(x^{n}|x_{i}^{n-1},y^{n}),$$

where $q(x^{n}|x_{i}^{n-1},y^{n})$ should be non‐zero whenever $p(x^{n}|x_{i}^{n-1})$ is. This step is completely general.

**Figure 2:**
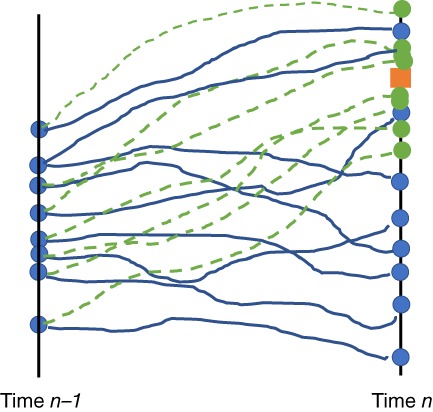
The proposal density. At time n − 1 we have a set of particles denoted by the filled circles. When we use the original model, they are propagated along the blue lines to time n. Because their distance to the observation (the box) varies significantly, so will their weights. When a proposed model is used, the particles at time n − 1 propagate along the green dashed lines and end up much closer to the observations. This leads to much more similar likelihood weights. However, because we have changed the model equations, the particles now also have proposal weights [Colour figure can be viewed at wileyonlinelibrary.com]

Now realise that drawing from $p(x^{n}|x_{i}^{n-1})$ corresponds to running the original stochastic model. We could instead draw from $q(x^{n}|x_{i}^{n-1},y^{n})$, which would correspond to a model equation from our choosing. Figure 2 illustrates the basic idea.

For instance when the original model is given by Equation 2, we can use

(16) $$x^{n}=g(x^{n-1},y^{n})+{\hat{\beta }}^{n},$$

in which *g*(.,.) is now the deterministic part and ${\hat{\beta }}^{n}$ is the stochastic part. These can be freely chosen, and examples of these will be given below. Note that we allowed *g*(..) to depend on the observations at the future time. This means that we generate the prior particles at time *n* by making one draw from $q(x^{n}|x_{i}^{n-1},y^{n})$ for each *i* where

(17) $$q(x^{n}|x^{n-1},y^{n})=p_{\hat{\beta }}\left\{x^{n}-g(x^{n-1},y^{n})\right\}.$$

In general, we draw the particles at time *n* from the alternative model *q*(**x** ^*n*^|**x** ^*n* − 1^,**y** ^*n*^) and account for this by changing the weights of the particles. Equations 14 and 15 can be written as

(18) $$p(x^{n})=\overset{N}{\underset{i=1}{\sum }}{\hat{w}}_{i}^{n-1}q(x^{n}|x_{i}^{n-1},y^{n})$$

and

(19) $$p(x^{n}|y^{n})=\overset{N}{\underset{i=1}{\sum }}{\hat{w}}_{i}^{n}q(x^{n}|x_{i}^{n-1},y^{n}),$$

where the weights are given by:

(20) $${\hat{w}}_{i}^{n-1}\propto w_{i}^{n-1}\frac{p(x_{i}^{n}|x_{i}^{n-1})}{q(x_{i}^{n}|x_{i}^{n-1},y^{n})}$$

and

(21) $${\hat{w}}_{i}^{n}\propto {\hat{w}}_{i}^{n-1}\frac{p(y^{n}|x_{i}^{n})}{p(y^{n})}\propto w_{i}^{n-1}p(y^{n}|x_{i}^{n})\frac{p(x_{i}^{n}|x_{i}^{n-1})}{q(x_{i}^{n}|x_{i}^{n-1},y^{n})}.$$

Here the coefficients of proportionality ensure that the weights sum to 1. In a reinterpretation of these equations, if $x_{i}^{n}$ is drawn from the alternative model $q(x^{n}|x_{i}^{n-1},y^{n})$ we can also write

(22) $$p(x^{n})\approx \overset{N}{\underset{i=1}{\sum }}{\hat{w}}_{i}^{n-1}\delta (x^{n}-x_{i}^{n})$$

and

(23) $$p(x^{n}|y^{n})\approx \overset{N}{\underset{i=1}{\sum }}{\hat{w}}_{i}^{n}\delta (x^{n}-x_{i}^{n}).$$

**Figure 3:**
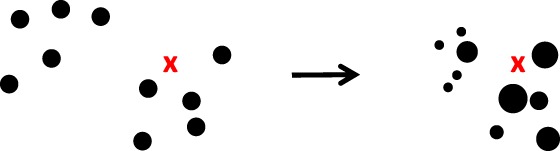
The typical proposal‐density particle filter. Left: the prior particles at time n − 1 (dots), with one observation, denoted by the red cross. Right: the posterior particles at time n, the larger the dot the larger its weight. Note that the particles do move in state space compared to a pure model propagation over one time step, and their weight contains contributions from the likelihood and from that movement [Colour figure can be viewed at wileyonlinelibrary.com]

We see that the weights now contain two factors, the likelihood weight, which also appears in the standard particle filter, and a proposal weight. These two weights have opposing effects. If we use a proposal density that strongly pushes the model towards the observations, the likelihood weight will be large because the difference between observations and model states becomes smaller, but the proposal weight becomes smaller because the model is pushed away from where it wants to go, so $p(x^{n}|x_{i}^{n-1})$ will be small. On the other hand, a weak pushing towards the observations keeps the proposal weight high, but leads to a small likelihood weight. This suggests that there is an optimum weight related to an optimal position $x_{i}^{n}$ for each particle as function of its position at time *n* − 1. This will be explored in equal‐weight formulations of the particle filter. Figure 3 shows how typical proposal‐density particle filters work. Equal‐weight particle filters are discussed later.

### 2.1 A simple relaxation scheme

To illustrate the idea of a proposal density, we consider the following simple example. We could add a relaxation or nudging term to the original equation to steer the particles towards the observations and make their weights more similar, as pioneered by van Leeuwen (2010) for geoscience applications. The model equation is written as:

(24) $$x^{m}=f(x^{m-1})+T\{y^{n}-H(x^{m-1})\}+{\hat{\beta }}^{m},$$

where we used time index *m* for the state vector to emphasise that there are several model time steps between observation times. **T** is a relaxation matrix of our choice. In this example, the deterministic part consists of the first two terms on the right‐hand side of the equation, while the third term denotes the random part. Let us assume the pdf of the random forcing is Gaussian with mean zero and covariance $\hat{Q}$. Then we can immediately write for the proposal density

(25) $$q(x^{m}|x^{m-1},y^{n})=N\left(f(x^{m-1})+T\{y^{n}-H(x^{m-1})\},\hat{Q}\right)$$

since the pdf of **x** ^*m*^ is just a shift in the mean of the pdf of ${\hat{\beta }}^{m}$. For the original model, we assume that the random part is Gaussian with zero mean and covariance **Q**, so that

(26) $$p(x^{m}|x^{m-1})=N\left(f(x^{m-1}),Q\right).$$

The change in the model equations is compensated for in particle filters by a change in the relative weight of each particle, and the expression for this change in weight for this case is:

(27) $$\begin{array}{lll} w_{i}^{m} & = & w_{i}^{m-1}\frac{p(x_{i}^{m}|x_{i}^{m-1})}{q(x_{i}^{m}|x_{i}^{m-1},y^{n})}\\ & \propto & w_{i}^{m-1}\frac{\exp\left[-J_{p}\right]}{\exp\left[-J_{q}\right]} \end{array}$$

in which, for Gaussian model errors,

(28) $$J_{p}=\frac{1}{2}{\left\{x_{i}^{m}-f(x_{i}^{m-1})\right\}}^{T}Q^{-1}\left\{x_{i}^{m}-f(x_{i}^{m-1})\right\}$$

and

(29) $$\begin{array}{lll} J_{q} & = & \frac{1}{2}{\left[x_{i}^{m}-f(x_{i}^{m-1})-T\{y^{n}-H(x^{m-1})\}\right]}^{T}\cdot \\ & & \;{\hat{Q}}^{-1}\left[x_{i}^{m}-f(x_{i}^{m-1})-T\{y^{n}-H(x^{m-1})\}\right]\\ & = & \frac{1}{2}{\left({\hat{\beta }}_{i}^{m}\right)}^{T}{\hat{Q}}^{-1}{\hat{\beta }}_{i}^{m}. \end{array}$$

Note that the normalization factors of the Gaussians do not have to be calculated explicitly if we stipulate that the sum of the weights has to be equal to one. The scheme is depicted by Algorithm 3.


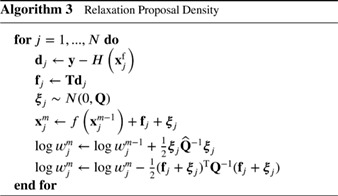


Simple as the scheme is, it does not solve the degeneracy problem. However, it can be used as a simple scheme when several model time steps are used between observation times, because the proposal is independent of the proposal at other time steps. This scheme can easily be used in combination with other schemes that work at observation time, to be discussed next.

### 2.2 Weighted Ensemble Kalman Filter

One could also use other existing data assimilation methods in proposal densities, like EnKFs or variational methods. In the Weighted Ensemble Kalman Filter (WEKF; Papadakis *et al.* 2010) the stochastic EnKF of Burgers *et al.* (1998) is used as follows. The EnKF update can be written as:

(30) $$x_{i}^{n}=x_{i}^{f}+K(y^{n}-Hx_{i}^{f}-\epsilon_{i})$$

in which $x_{i}^{f}=f(x_{i}^{n-1})+\beta_{i}^{n}$, the matrix *K* is the ensemble Kalman gain and ***ϵ***_*i*_∼*N*(0,**R**), with **R** the observational error covariance. Using the expression for the forecast $x_{i}^{f}$ in the Kalman filter update equation, we find:

(31) $$x_{i}^{n}=f(x_{i}^{n-1})+K\left\{y^{n}-Hf(x_{i}^{n-1})\right\}+(I-KH)\beta_{i}^{n}-K\epsilon_{i},$$

which we can rewrite as the sum of a deterministic and a stochastic part as:

(32) $$x^{n}=g(x^{n-1},y^{n})+{\hat{\beta }}_{i}^{n}$$

identifying $g(x^{n-1})=f(x_{i}^{n-1})+K\left\{y^{n}-Hf(x_{i}^{n-1})\right\}$ and ${\hat{\beta }}_{i}^{n}=(I-KH)\beta_{i}^{n}-K\epsilon_{i}$. Therefore, we find for the proposal density:

(33) $$q(x^{n}|x_{i}^{n-1},y^{n})=N\left[f(x^{n-1})+K\{y^{n}-Hf(x^{n-1})\},\hat{Q}\right]$$

with

(34) $$\hat{Q}=(I-KH)Q{\left(I-KH\right)}^{T}+KRK^{T}.$$

Strictly speaking, this is correct only if the Kalman gain is calculated using the ensemble covariance of *f*(**x** ^*n* − 1^), so without the model errors ***β*** ^*n*^, otherwise the proposal is not Gaussian. We can calculate the weights of the particles in a similar way to the previous example. Algorithm 4 shows the algorithmic steps.


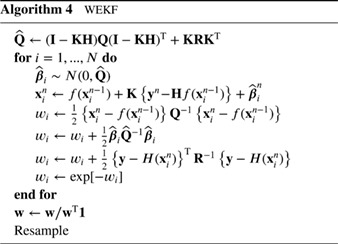


The behaviour of this filter has been studied extensively by Morzfeld *et al.* (2017). In high‐dimensional systems, this filter will be degenerate, consistent with the theory of Snyder *et al.* (2015), and as proven in the next section. The only way to make this work is to include localization, not only at the EnKF level, but also at the level of the particle filter (e.g. Morzfeld *et al.* 2017).

### 2.3 Optimal proposal density

In the class of particle filters in which the proposal density of each particle is dependent only on that particle, an optimal proposal density can be derived, as in e.g. Doucet *et al.* (2001). They defined optimality as the proposal density that gives a minimal variance of the weights, and Snyder *et al.* (2015) provide an elegant proof of this optimality. In this section we generalize this result and show that the optimal proposal density is optimal even when each particle has its own proposal density which is allowed to depend on all previous particles, so a proposal of the form $q(x^{n}|i,x_{1:N}^{n-1},y^{n})$.

Snyder *et al.* (2015) concentrate on the case that one is interested in – an optimal representation of *p*(*x* ^*n*^,*x* ^*n* − 1^|*y* ^*n*^) in a sequential algorithm – so in a sequential smoother. To this end they introduce the random variable

(35) $$w^{*}(x^{n},x^{n-1})=\frac{p(x^{n},x^{n-1}|y^{n})}{q(x^{n},x^{n-1}|y^{n})}$$

and determine that proposal density *q* that minimizes the variance in the weights *w* ^∗^, with the expectation taken over the density from which we draw the particles, so the proposal *q*.

Here we show that the optimal proposal density is also optimal for the strict filtering case, so when we are interested in minimal variance of the weights at time *n* only. Specifically, the question is: given the set of particles at *t* = *n* − 1 drawn from *p*(**x**_*n* − 1_|**y**_1:*n* − 1_), which proposal density of the form $q(x^{n}|i,x_{1:N}^{n-1},y^{n})$ gives minimal variance of the weights at time *n*?

Using Bayes' formula, we can write the expression for the weight of particle *i* as function of the state at time *n* as:

(36) $$\begin{array}{lll} w_{i}^{n}=w_{i}(x_{i}^{n}) & = & \frac{p(y^{n}|x_{i}^{n})}{Np(y^{n})}\frac{p(x_{i}^{n}|x_{i}^{n-1})}{q(x_{i}^{n}|i,x_{1:N}^{n-1},y^{n})}\\ & = & \frac{p(y^{n}|x_{i}^{n-1})}{Np(y^{n})}\frac{p(x_{i}^{n}|x_{i}^{n-1},y^{n})}{q(x_{i}^{n}|i,x_{1:N}^{n-1},y^{n})}, \end{array}$$

where we assume, without loss of generality, an equally weighted ensemble at time *n* − 1. Note that the second equality follows from Bayes' theorem, as follows:

(37) $$\begin{array}{lll} p(x_{i}^{n}|x_{i}^{n-1},y^{n}) & = & \frac{p(y^{n}|x_{i}^{n},x_{i}^{n-1})}{p(y^{n}|x_{i}^{n-1})}p(x_{i}^{n}|x_{i}^{n-1})\\ & = & \frac{p(y^{n}|x_{i}^{n})}{p(y^{n}|x_{i}^{n-1})}p(x_{i}^{n}|x_{i}^{n-1}). \end{array}$$

Consider the pair of random variables (*I*,**X** ^*n*^) where Prob(*I* = *i*) = 1/*N* and, conditionally on *I* = *i*, $X^{n}∼q(x^{n}|i,x_{1:N}^{n-1},y^{n})$. Furthermore, define the associated random variable

(38) $$W=w_{I}(X^{n})=\frac{p(y^{n}|x_{I}^{n-1})}{Np(y^{n})}\frac{p(X^{n}|x_{I}^{n-1},y^{n})}{q(X^{n}|I,x_{1:N}^{n-1},y^{n})},$$

where

(39) $$p(y^{n})=\frac{1}{N}\overset{N}{\underset{j=1}{\sum }}p(y^{n}|x_{j}^{n-1}).$$

In order to find the proposal *q* that minimizes the variance of *W*, we use the well‐known law of total variance (derived in the Appendix for completeness):

(40) $${\mathrm{var}}_{W}(W)={\mathrm{var}}_{I}\{E_{X^{n}|I}(W)\}+E_{I}\{{\mathrm{var}}_{X^{n}|I}(W)\}.$$

First, we see that, under the proposal *q*,

(41) $$E_{X^{n}|I}(W)=\frac{p(y^{n}|x_{I}^{n-1})}{Np(y^{n})}\int p\left(x^{n}|x_{I}^{n-1},y^{n}\right)dx^{n}=\frac{p(y^{n}|x_{I}^{n-1})}{Np(y^{n})}$$

is independent of *q*. Moreover, $E_{W}(W)=E_{I}\{E_{X^{n}|I}(W)\}=1/N$ and thus the first term in var_*W*_(*W*) is

(42) $$\frac{1}{N}\underset{i}{\sum }\frac{p{\left(y^{n}|x_{i}^{n-1}\right)}^{2}}{N^{2}p{\left(y^{n}\right)}^{2}}-\frac{1}{N^{2}}=\frac{1}{N}\underset{i}{\sum }{\left(\frac{p(y^{n}|x_{i}^{n-1})}{Np(y^{n})}-\frac{1}{N}\right)}^{2}\geq 0.$$

For the second term we use that ${\mathrm{var}}_{X^{n}|I}(W)\geq 0$ with equality if and only if *W* is almost surely constant in **X** ^*n*^, that is if and only if

(43) $$\frac{p(x^{n}|x_{i}^{n-1},y^{n})}{q(x^{n}|i,x_{1:N}^{n-1},y^{n})}=\text{cst}(i,x_{1:N}^{n-1},y^{n}).$$

in which cst(..) is this constant which can depend on variables other than **x** ^*n*^. Because both *p* and *q* are densities (in **x** ^*n*^), *cst* = 1. Combining these results, we have a lower bound for var(*W*) that is determined by the variance of $p(y^{n}|x_{i}^{n-1})$ over *i*, with equality if and only if

(44) $$q(x^{n}|i,x_{1:N}^{n-1},y^{n})=p(x^{n}|x_{i}^{n-1},y^{n}).$$

Note that this is a new result, as previous proofs only considered proposal densities of the form $q(x^{n}|x_{i}^{n-1},y^{n})$, and we extended it to more general proposal densities of the form $q(x^{n}|i,x_{1:N}^{n-1},y^{n})$.

This remarkable result shows that firstly the optimal proposal density, so $p(x^{n}|x_{i}^{n-1},y^{n})$, does indeed lead to the lowest variance in the weights for the class of particle filters in which the transition density is of the form $q(x^{n}|i,x_{1:N}^{n-1},y^{n})$. Secondly, it shows that we can predict the variance in the weights without doing the actual experiment, for any number of particles, provided we can compute $p(y^{n}|x_{i}^{n-1})$, and thirdly the weights are *independent of the position of the particles* **x** ^*n*^. Unfortunately, this variance is zero only when the observations are not dependent on the state at time *n* − 1, which is never the case in the geosciences.

A simple case where we can compute both the optimal proposal density and the weights $p(y^{n}|x_{i}^{n-1})$ is when $p(x^{n}|x_{i}^{n-1})$ is given by Equation 11 and the observation operator *H* = **H** is linear. By the same argument that is used to derive the Kalman filter update, we find

(45) $$\begin{array}{ll} p(x^{n}|x_{i}^{n-1},y^{n}) & =N\left[f(x_{i}^{n-1})+T\{y^{n}-Hf(x_{i}^{n-1})\},\right.\;\\ & \;\times \left.(I-TH^{T})Q\right],\; \end{array}$$

where **T** = **QH** ^T^(**HQH** ^T^ + **R**)^−1^ is the Kalman‐like gain with the background covariance **Q**, and the weights are proportional to

(46) $$p(y^{n}|x_{i}^{n-1})=N\left\{Hf(x_{i}^{n-1}),HQH^{T}+R\right\}.$$

This shows two things. First, in this special case, the simple relaxation scheme of Section 2.1 is equal to the optimal proposal when the relaxation matrix **T** is chosen as above. Second, comparing the weights of the optimal proposal with the weights of the standard filter, they both depend on the squared distance $||y^{n}-Hf(x_{i}^{n-1})|{|}^{2}$, and $||y^{n}-Hx_{i}^{n}|{|}^{2}$, respectively, but in the standard particle filter the distance is defined w.r.t. **R** and in the optimal proposal the distance it is defined is w.r.t. **HQH** ^T^ + **R**. Hence the weights with the optimal proposal are more similar, but the improvement is substantial only if **Q** is large, and the analysis of weight collapse by Snyder *et al.* (2008) still applies.

One can extend the optimal proposal density idea to more than one time step. Snyder *et al.* (2015) show that the optimal proposal is the proposal of this form with minimal variance in the weights in this case too, which can also easily be seen by applying the above to

$$W=w_{i}(x^{n})=\frac{p(y^{n}|x_{i}^{m-1})}{Np(y^{n})}\frac{p(x^{n}|x_{i}^{m-1},y^{n})}{q(x^{n}|x_{i}^{m-1},y^{n})}$$

for *m* < *n*.

Looking back at the filters described in the previous sections, we find the following. The relaxation scheme uses a simple proposal density that is of the form $q(x^{n}|x_{i}^{n-1},y^{n})$, so the theory holds, and that proposal will lead to degenerate results. This is indeed the finding of van Leeuwen (2010). The WEKF has a proposal that depends on all particles at time *n* − 1 through the Kalman gain **K**, so the proposal is of the form $q(x^{n}|i,x_{1:N}^{n-1},y^{n})$. Hence also this filter will perform worse than the optimal proposal and hence will be degenerate for high‐dimensional systems. This was first explored in detail by Morzfeld *et al.* (2017).

### 2.4 Implicit Particle Filter

The Implicit Particle Filter is an indirect way to draw from the optimal proposal, even over several time steps. Often the assumption is made that the model errors of both original model and proposal density are Gaussian, and the observation operator **H** is linear. In this case, a draw from the optimal proposal is a draw from a multivariate Gaussian, and we know how to do that.

**Figure 4:**
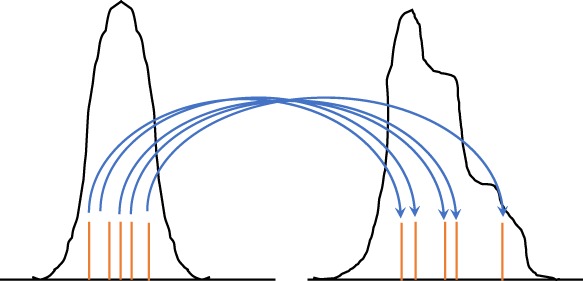
The Implicit Particle Filter. Samples (red bars in the left pdf) are drawn from the standard multivariate Gaussian and transformed via Equation 49 to weighted samples from the posterior (red bars in the right pdf) [Colour figure can be viewed at wileyonlinelibrary.com]

However, when **H** is nonlinear, or when the proposal is used over several model time steps, the density to draw from is not now Gaussian. Chorin *et al.* (2010) realised that one could still draw from a Gaussian and then apply a transformation to that draw to find samples from the optimal proposal density. The method is explained here for one time step, but the extension to multiple time steps is straightforward. Figure 4 illustrates the basic idea.

As mentioned in Section 2 on the proposal density, the posterior pdf can be written as

(47) $$p(x^{n}|y^{n})=\overset{N}{\underset{i=1}{\sum }}w_{i}^{n-1}\frac{p(y^{n}|x^{n})}{p(y^{n})}\frac{p(x^{n}|x_{i}^{n-1})}{q(x^{n}|x_{i}^{n-1},y^{n})}q(x^{n}|x_{i}^{n-1},y^{n}).$$

The scheme draws from a Gaussian proposal *q*(***ξ***) = *N*(0,**I**), and we can write the transformation as $q(x^{n}|x_{i}^{n-1},y^{n})=q(\xi )J_{i}^{-1}$ in which **J**_*i*_ is the Jacobian of the transformation from **x** ^*n*^ to ***ξ***. That transformation is found implicitly, hence the name of the filter, by defining

(48) $$F_{i}(x^{n})=-\log\left[p(y^{n}|x^{n})p(x^{n}|x_{i}^{n-1})\right]$$

and, after drawing ***ξ***_*i*_ for each particle, solving for **x** ^*n*^ in

(49) $$F_{i}(x^{n})=\frac{1}{2}\xi_{i}^{T}\xi_{i}+\varphi_{i}$$

for each particle, in which $\varphi_{i}=\min_{x^{n}}F_{i}(x^{n})\propto p(y^{n}|x_{i}^{n-1})$. The weights of the particles become

(50) $$\begin{array}{lll} w_{i}^{n} & = & w_{i}^{n-1}\frac{p(y^{n}|x_{i}^{n})}{p(y^{n})}\frac{p(x_{i}^{n}|x_{i}^{n-1})}{q(x_{i}^{n}|x_{i}^{n-1}y^{n})}\\ & = & w_{i}^{n-1}\frac{\exp\left[-F_{i}(x_{i}^{n})\right]}{\exp\left[-\frac{1}{2}\xi_{i}^{T}\xi_{i}\right]}J_{i}\\ & = & w_{i}^{n-1}\frac{\exp\left[-F_{i}(x_{i}^{n})\right]}{\exp\left[-F_{i}(x^{n})+\varphi_{i}\right]}J_{i}\\ & = & w_{i}^{n-1}\exp\left[-\varphi_{i}\right]J_{i}. \end{array}$$

Interestingly, while the optimal proposal density shows that the weights are only dependent on the position of the particles at the previous time, so on $x_{i}^{n-1}$ via *φ*_*i*_, the implicit map makes the weights also dependent on the positions at the current time *n*, so on $x_{i}^{n}$ via the Jacobian of the transformation between ***ξ*** and **x**. Only when the Jacobian is a constant, so when *F*_*i*_ is quadratic in **x**_*i*_, this dependence disappears.

Solving Equation 49 is not straightforward in general. Morzfeld *et al.* (2012) suggest a random map of the form

(51) $$x_{i}^{n}=x_{i}^{a}+\lambda_{i}(\xi_{i})P^{1/2}\xi_{i},$$

in which **P** is a chosen covariance matrix, ideally the covariance of the posterior pdf, $x_{i}^{a}=\arg\min F_{i}(x^{n})$ and *λ*_*i*_ is a scalar. This transforms the problem into solving a highly nonlinear scalar equation for *λ*_*i*_, which is a much simpler problem than finding $x_{i}^{n}$ directly. This map can be shown to be a bijection when $F_{i}(x_{i}^{n})$ has only closed contours in the high‐probability regions; otherwise one would first have to choose a closed contour area and then perform the map. In general, when the optimal proposal (over several time steps if needed) is multimodal, the transformation from the state variable to a Gaussian is not monotonic, and the Implicit Particle Filter needs to be adapted, for example, by using a separate Gaussian for each mode. The algorithm is given in Algorithm 5.


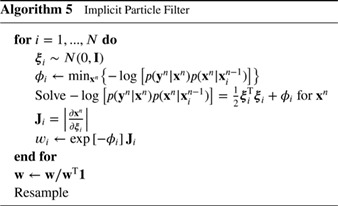


Of further interest is that $x_{i}^{a}$ is the same as the solution to a 4D‐Var problem well known in meteorology. But it is a special 4D‐Var as the initial position of each particle is fixed and it has to be a weak‐constraint 4D‐Var. The latter condition is needed as a strong‐constraint 4D‐Var would have no possibility to move a particle in state space as its initial condition is fixed.

However, this filter will also suffer from weight collapse in high‐dimensional applications as it is still a sampling scheme for the optimal proposal density. The following sections will discuss ways to improve on the optimal proposal.

### 2.5 Equal weights by resampling at time n − 1

As noted already in Equation 36, we can write Equation 13 as

(52) $$\begin{array}{lll} p(x^{n}|y^{n}) & = & \overset{N}{\underset{i=1}{\sum }}\frac{w_{i}^{n-1}p(y^{n}|x_{i}^{n-1})}{p(y^{n})}p(x^{n}|x_{i}^{n-1},y^{n})\\ & = & \overset{N}{\underset{i=1}{\sum }}\alpha_{i}p(x^{n}|x_{i}^{n-1},y^{n}), \end{array}$$

where

(53) $$\alpha_{i}=\frac{w_{i}^{n-1}p(y^{n}|x_{i}^{n-1})}{p(y^{n})}.$$

This says that, assuming the pdf at the previous time can be approximated by a set of *N* particles, the analysis distribution is a mixture of the optimal proposal pdfs $p(x^{n}|x_{i}^{n-1},y^{n})$ with mixture weights *α*_*i*_.

If we can compute the optimal proposal density and the weights *α*_*i*_ in closed form, we can also draw samples *directly from this mixture density*. For this, we first draw an index *I* from the discrete distribution with weights *α*_*i*_, Prob(*I* = *j*) = *α*_*j*_, followed by a draw from the corresponding pdf $p(x^{n}|x_{i}^{n-1},y^{n})$. Doing this *N* times will lead to *N* different particles with equal weights because each of them is an independent draw directly from the posterior. If the index *I* is equal to a value *j* more than once, the particle $x_{j}^{n-1}$ is propagated from time *n* − 1 to time *n* with independent random forcing for each of these draws. This simple scheme provides better samples than the optimal proposal density because all particles are different at time *n* by construction.

However, this does not solve the problem of weight collapse because drawing the index *I* is nothing other than resampling the particles at time *n* − 1 with weights proportional to $w_{i}^{n-1}p(y^{n}|x_{i}^{n-1})$. If $w_{i}^{n-1}=1/N$, the variance of these weights is exactly equal to the lower bound that we found in Section 2.3. The main difference is that the collapse now happens at time *n* − 1. The only advantage is that all particles will be different at time *n*.

If we cannot compute the optimal proposal density and the weights *α*_*i*_ in closed form, we can still use the importance sampling idea to draw from the mixture *p*(**x** ^*n*^|**y** ^*n*^) by drawing pairs (*I*,**X** ^*n*^) consisting of an index *I* and a state **X** ^*n*^ at time *n*. We choose a proposal distribution $\beta_{i}=\beta_{i}(y^{n})$ for the index and proposal distributions $q(x^{n}|x_{i}^{n-1},y^{n})$ for the state. Then we draw the index *I*_*i*_ with $\text{Prob}(I_{i}=j)=\beta_{j}(y^{n})$ and conditionally on *I*_*i*_ = *j* we draw $x_{i}^{n}$ from $q(x^{n}|x_{j}^{n-1},y^{n})$. Finally, we compute weights $w_{i}^{n}$ by

$$w_{i}^{n}\propto \frac{w_{j}^{n-1}p(x_{i}^{n}|x_{j}^{n-1})p(y|x_{i}^{n})}{\beta_{j}(y^{n})q(x^{n}|x_{j}^{n-1},y^{n})}\;\text{if}\;I_{i}=j.$$

The particles $x_{i}^{n}$ with weights $w_{i}^{n}$ provide the desired approximation of *p*(**x** ^*n*^|**y** ^*n*^) whereas the indices *I*_*i*_ can be discarded after the weights have been computed. We could produce an evenly weighted approximation by a further resampling step, or take the weights $w_{i}^{n}$ into account during the next iteration.

In this approach we can obtain equal weights $w_{i}^{n}$ by choosing

$$q(x^{n}|x_{j}^{n-1},y^{n})=p(x^{n}|x_{j}^{n-1},y^{n})$$

and

$$\beta_{i}(y^{n})\propto w_{i}^{n-1}p(y^{n}|x_{i}^{n-1}).$$

With this choice, we draw directly from the mixture Equation 52. As mentioned before, although the weights $w_{i}^{n}$ are then equal to 1/*N*, the algorithm contains a hidden weighting and resampling step of particles at time *n* − 1. It thus remains susceptible to weight collapse in high dimensions.

This approach of using importance sampling for the joint distribution of (*I*,**X** ^*n*^) is due to Pitt and Shephard (1999) who called it *Auxiliary Particle Filter* (the index *I* is an auxiliary variable that is discarded at the end). They discuss, in addition, approximations of the optimal proposal density and the optimal weights *α*_*i*_. One of their suggestions is to use for the index *I* the proposal with weights

$$\beta_{i}\propto w_{i}^{n-1}p(y^{n}|\mu_{i}^{n}),$$

where $\mu_{i}^{n}$ is a likely value of the distribution $p(x^{n}|x_{i}^{n-1})$; for example, the mean or median or simply a draw from it. Typically, $\mu_{i}^{n}$ is found by a probing step where particles at time *n* are propagated by a simplified model, for example, by omitting stochastic terms or with simplified subgrid‐scale parametrizations or thermodynamics. If *I*_*i*_ = *j* and the state $x_{i}^{n}$ at time *n* is proposed from $p(x^{n}|x_{j}^{n-1})$, the weights become

$$w_{i}^{n}\propto \frac{p(y|x_{i}^{n})}{p(y^{n}|\mu_{j}^{n})}.$$

They will vary less provided $x_{i}^{n}$ is close to $\mu_{j}^{n}$, i.e. provided the simplified model is a good approximation to the full model and the stochastic part of the full model is small.

### 2.6 Equivalent‐Weights Particle Filter

The EWPF (van Leeuwen, 2010; Ades and van Leeuwen, 2013) uses the idea to obtain a more evenly weighted set of particles by not sampling from the exact posterior, but allowing for a small error. It starts with determining the weight of each particle at the mode of $p(x^{n}|x_{i}^{n-1},y^{n})$ for each particle *i*, $w_{i}^{\max}\propto p(y^{n}|x_{i}^{n-1})$. Note that these weights are equal to the weights obtained in the optimal proposal density. In the optimal proposal density case, the weights do not depend on the position **x** ^*n*^ of the particle, but note that the proposal used here will be different.

The particles are not moved to these modes, but the weights are used to define a target weight. This target weight *w*_target_ is chosen such that a certain fraction *ρ* of particles can reach that weight. To this end we sort the weights in magnitude from high to low in an array $w_{i}^{*},\;i=\{1,2,...,N]\}$ and set $w_{\text{target}}=w_{N*\rho }^{*}$. For instance, with 100 particles and a fraction of *ρ* = 0.8, we would find $w_{\text{target}}=w_{80}^{*}$.

The next step is to find a position in state space for each particle that can reach this weight such that its weight is exactly equal to the target weight. This means we solve for **x** ^*n*^ in

(54) $$w_{i}(x^{n})=w_{\text{target}}$$

for each particle *i* that can reach this weight. There are many solutions of this equation, but we choose the one which is on the line through $x_{i}^{a}$ and $f(x_{i}^{n-1})$ and is closest to $f(x_{i}^{n-1})$. Denote this position as $x_{i}^{*}$. Note that this is a purely deterministic move, so a stochastic part still has to be added. The final position of these particles is then determined by adding a very small random perturbation ***ξ*** from a chosen density, so

(55) $$x_{i}^{n}=x_{i}^{*}+\xi_{i}^{n}.$$

This stochastic move ensures that the proposal has full support and is not a delta function centred at $x_{i}^{*}$. The density of ***ξ***_*i*_ should on the one hand have most of its mass concentrated around 0 in order not to change the weights of the particles too much, and on the other hand it should be relatively constant since we divide by the value of the proposal density. Both requirements cannot be fulfilled exactly, but we can take some error in the sampling into account and choose a narrow uniform distribution. The scheme is depicted in Algorithm 6 for the special case of Gaussian model errors and a linear observation operator. If these conditions do not hold, one will typically need iterations to solve for *a*_*i*_ and *b*_*i*_.


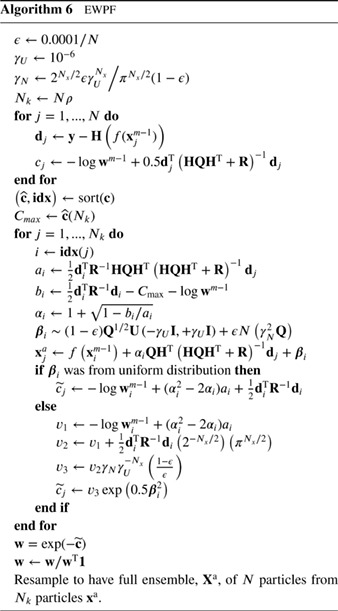


It is common knowledge (e.g. Doucet *et al.* 2001), that the proposal should be wider or at least as wide as the target, while the width of the stochastic part of the proposal is chosen very small here. The reason that we can do this is that the position of the centres of these proposal densities are typically further away from the observations than, for example, in the optimal proposal because the target weight forces particles away from their optimal positions, so away from the observations. This means that the deterministic moves of the particles ensure a large spread in the full proposal.

A formal way to avoid such an error has been described by Ades and van Leeuwen (2015b). They choose the proposal to be a mixture of a uniform density and a Gaussian which is also used in Algorithm 6. Both have small variance, and the mixture coefficient of the uniform density is chosen to be much larger than that of the Gaussian. This means that drawing from the Gaussian and also drawing from its tails becomes highly unlikely. In practice, since we always work with small ensemble sizes, the chance of filter degeneracy by drawing from the Gaussian, and then drawing from the tail of the Gaussian, is indeed highly unlikely.

Finally, the full weights for the new particles are calculated and the whole ensemble is resampled, including those particles that were unable to reach the target weight. Because of the target‐weight construction, the weights of the particles are very similar, and filter degeneracy is avoided. This filter has been used in a reduced‐gravity ocean model by Ades and van Leeuwen (2015b), and in the same system studied for the gravity‐wave production by the scheme in Ades and van Leeuwen (2015a). It has also been applied in a climate model by Browne and van Leeuwen (2015).

To analyse the scheme further, we can look again at the variance of the weights. For this it is important to note that this scheme does not see the weight of a particle as a function of the state **X** and particle index *I*, but rather the state as function of the weight *W* and index *I*, so **X**(*W*,*I*). Specifically, *W*|*I* has values in two ranges. For the particles with *I* = *i* that can reach the target weight, we find *w*|*I* = *w*_*target*_ + *ϵ*_*i*_ in which *ϵ*_*i*_ is a small perturbation from the target weight due to the small stochastic move discussed above. For those particles that cannot reach the target weight, their weights are very close to zero. So we find

(56) $$E_{I}[W]\approx \rho (w_{\text{target}}+\bar{\epsilon })+(1-\rho )0=\rho (w_{\text{target}}+\bar{\epsilon }),$$

in which $\bar{\epsilon }=E_{I}[\epsilon ]$. If *H* is linear and the errors in the observations and the model equations are Gaussian, we find $\bar{\epsilon }=0$, but if any of these three conditions does not hold this is not necessarily so. However, we do know that by construction $|\bar{\epsilon }|<<1$. Since the sum of the weights should be equal to 1, we find that *w*_target_≈1/(*Nρ*), and hence *E*_*I*_[*W*] = 1/*N*, as expected. Furthermore

(57) $$\begin{array}{lll} {\mathrm{var}}_{I}(W) & = & \rho \overset{\rho N}{\underset{i=1}{\sum }}{\left(w_{\text{target}}+\epsilon_{i}\right)}^{2}-{\left(\rho w_{\text{target}}\right)}^{2}\\ & \approx & \frac{1}{N^{2}}\frac{1-\rho }{\rho }. \end{array}$$

This expression shows that the variance in the weights ranges between 0 for *ρ* = 1, so when all particles are kept, to (*N* − 1)/*N* ^2^≈1/*N* for *ρ* = 1/*N*, so when one particle is kept. We can compare this with the optimal proposal when the number of independent observations is large. In that case one particle will have a weight very close to one, and the rest will have weights very close to zero. The variance in the weights is then (*N* − 1)/*N* ^2^≈1/*N*, indeed equal to the *ρ* = 1/*N* case in the EWPF scheme, as expected. However, the EWPF can reduce that variance, even to zero, depending on the choice of the tuning parameter *ρ*.

When this tuning parameter is chosen close to one, the target weight will be low, and hence particles will be moved further away from the mode of the optimal proposal density. In practice this means that the particles are pushed further away from each other, leading to a wider posterior pdf. A small value for the fraction will have the opposite effect. Since we do not know *apriori* what the width of the posterior should be, this is a clear drawback of this method. We will come back to this later.

### 2.7 Implicit Equal‐Weights Particle Filter

In the IEWPF we set the target weight equal to the minimum of the optimal proposal weights for all particles. Then, the position of each particle is set to the mode of the optimal proposal density plus a scaled random perturbation. The scale factor is chosen such that the weight of each particle is equal to the target weight. Note that in the standard setting, no resampling is needed, but Zhu *et al.* (2016) gives other possibilities.

The implicit part of the scheme follows from drawing samples implicitly from a standard Gaussian distributed proposal density *q*(***ξ***) instead of the original *q*(**x** ^*n*^|**x** ^*n* − 1^,**y** ^*n*^), following the same procedure as in the Implicit Particle Filter. We define a relation

(58) $$x_{i}^{n}=x_{i}^{a}+\alpha_{i}^{1/2}P^{1/2}\xi_{i}^{n},$$

where $x_{i}^{a}$ is the mode of $p(x^{n}|x_{i}^{n-1},y^{n})$, **P** is a measure of the width of that pdf, $\xi_{i}^{n}\in {ℜ}^{N_{x}}$ is a standard Gaussian‐distributed random vector, and *α*_*i*_ is a scalar.

The IEWPF scheme is different from the Implicit Particle Filter in that it chooses the *α*_*i*_ such that all particles get the same weight *w*_target_, so the scalar *α*_*i*_ is determined for each particle from:

(59) $$w_{i}(\alpha_{i})=\frac{p(x_{i}^{n}|x_{i}^{n-1},y^{n})p(y^{n}|x_{i}^{n-1})}{Np(y^{n})q(x^{n}|i,x_{1:N}^{n-1},y^{n})}=w_{\text{target}}.$$

This target weight is equal to the lowest weight over all particles in an optimal proposal. This ensures that the filter is not degenerate in systems with arbitrary dimensions and an arbitrary number of independent observations. The resulting equation for each *α*_*i*_ is nonlinear and complex because it will contain the Jacobian of the transformation from ***ξ*** ^*n*^ to **x** ^*n*^, similar to the Implicit Particle Filter. The Jacobian will contain the derivative of *α*_*i*_ to ***ξ***_*i*_, which is the main source of the complexity in this scheme. Algorithm 7 depicts the scheme for the case of a linear observation operator. A nonlinear observation operator will lead to more complicated equations for the *α*s.


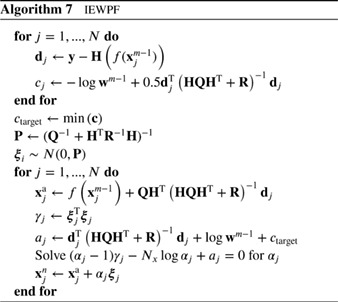


The scheme is similar to the optimal proposal density using the Implicit Particle Filter by first determining the mode of the proposal and then adding a random vector. The difference is that in the IEWPF the size of the vector is determined such that the each particle reaches the target weight. It turns out that this construction excludes part of state space for all but one particle. For each particle the excluded part is different, so the ensemble samples the whole space, but the individual particles do not. Details of the method can be found in Zhu *et al.* (2016).

**Figure 5:**
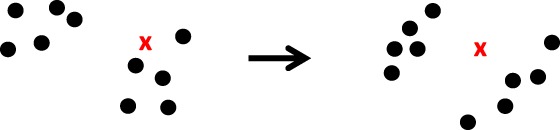
The implicit equal‐weights particle filter. Left: the prior particles at time n − 1(dots), with one observation, denoted by the red cross. Right: the posterior particles. Note that the weights are equal, but some particles have moved away from the observations to ensure equal weights [Colour figure can be viewed at wileyonlinelibrary.com]

Analysing the scheme in more detail, the proposal density used in this scheme is of one dimension lower than that of the state itself. The *direction* of the random vector in state space is determined by the proposal density, but the *size* of the random vector is then found deterministically, dependent on that direction. So the proposal density misses one degree of freedom for all but one particle – the particle with the lowest weight that has *α*_*i*_ = 1. Although missing one degree of freedom in a very high‐dimensional system might seem acceptable, it does lead to a bias. Figure 5 shows how the implicit equal‐weights particle filter works.

### 2.8 Discussion

We first note that the optimal proposal is only optimal in a very limited sense, as has been known a long time with the invention of the auxiliary particle filter. We have seen that it is not difficult to generate particle filters that even have zero variance in the weights. In the optimal proposal setting, one forces Prob(*I* = *i*) = 1/*N*, while the simple choice $\text{Prob}(I=i)\propto p(y^{n}|x_{i}^{n-1})$ leads to an equal‐weight particle filter. Furthermore, schemes have been introduced that consider the state as a function of the state at the previous time and the weight the state at the current time should obtain, so instead of working with *W*(**X**,*I*) we choose **X**(*W*,*I*), which opens up a whole new range of efficient particle filters in high‐dimensional systems.

The EWPF and the IEWPF are by construction particle filters that are not degenerate in high‐dimensional systems and do not rely on localization. However, it is easy to see that both filters are biased, or inconsistent. In the limit of an infinite number of particles, the target‐weight constructions will prevent the schemes to converge to the full posterior pdf. The schemes are only of interest when the ensemble size is limited. As long as the bias from the target‐weight construction is smaller than the Monte‐Carlo error, this bias is of no direct consequence. It will be clear that the number of possible methods that have this property is huge, and much more research is needed to explore the best possibilities.

## 3 TRANSPORTATION PARTICLE FILTERS

In resampling particle filters, the prior particles are first weighted to represent the posterior and then transformed to unweighted particles simply by duplicating high‐weight particles and abandoning low‐weight particles. In transformation particle filters, one tries to find a transformation that moves particles from the prior to particles of the posterior in a deterministic manner. A related approach, which uses random transformation steps, is based on tempering the likelihood, which we also discuss in this section.

### 3.1 One‐step transportation

In one‐step transportation one tries to transform samples from the prior into samples from the posterior in one transformation step. An example is the Ensemble Transform Particle Filter (ETPF; Reich 2013), in which the unweighted particles are linear combinations of the weighted particles, so one writes

(60) $$X^{a}=X^{f}D,$$

in which the matrix $X^{f}=(x_{1}^{f},\text{…}\;,x_{N}^{f})$ and similar for **X** ^a^, and in which **D** is a transformation matrix. The only conditions on **D** are that *d*_*ij*_ ≥ 0, $\sum_{i}d_{ij}=1$ and $\sum_{j}d_{ij}=w_{i}N$. These three conditions leave a lot of freedom for all *N* ^2^ elements of *D*, and a useful way to determine them is to ensure minimal overall movement in state space of the particles from prior to posterior. This leads to an optimal transportation problem and is typically solved by minimizing a cost function that penalizes movement of particles.

We can see immediately that this method will not work when the weights are degenerate as the solution will be degenerate and all particles have no other choice than move to the prior particle with weight (close to) one. However, the strength of this filter is that it allows for localization in a very natural way by making the weights, and hence the matrix **D**, space dependent. The method will be discussed in more detail in Section 4 on localization. Here we provide the basic algorithm in Algorithm 8.


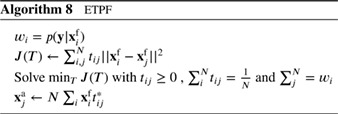


The ETPF provides a direct map from prior to posterior particles without explicitly constructing a transformation map. An alternative approach has been suggested in Moselhy and Marzouk (2012), where an approximate transportation map $\tilde{T}$ is constructed such that $\tilde{T}$ belongs to certain family of maps and $\tilde{T}$ is chosen such that the Kullbeck–Leibler divergence between the pdf generated by $\tilde{T}$ and the posterior pdf is minimized. Spantini *et al.* (2018) gives an efficient implementation in the context of filtering and smoothing for low‐dimensional systems.

### 3.2 Tempering of the likelihood

Instead of trying to transform the particles from the prior to particles from the posterior in one step, one can also make this a smoother transition. In tempering (Neal 1996, also Del Moral *et al.* 2006, Beskos *et al.* 2014) one factorizes the likelihood as follows:

(61) $$p(y|x)=p{\left(y|x\right)}^{\gamma_{1}}...p{\left(y|x\right)}^{\gamma_{m}},$$

with 0 < *γ*_*i*_ < 1 and ensuring that the sum of the *γ*s is equal to 1. Then the weighting of the particle filter is first done with the first factor, so

(62) $$p_{1}(x|y)=\frac{p{\left(y|x\right)}^{\gamma_{1}}}{p{\left(y\right)}^{\gamma_{1}}}p(x).$$

**Figure 6:**
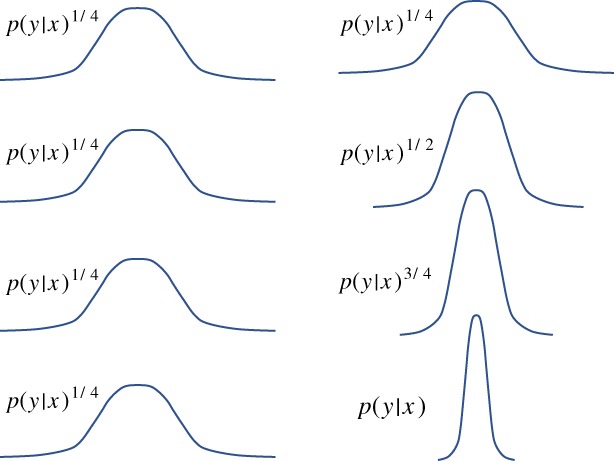
Tempering. The left‐hand side shows the tempered likelihood functions used in every iteration of the tempering scheme, so every particle filter update. We have chosen γ_i_ = 1/4 in this example. The right‐hand side illustrates how the full likelihood is built up during the tempering process [Colour figure can be viewed at wileyonlinelibrary.com]

The reason for this is that the likelihood is much less peaked, and hence the degeneracy can be avoided when *γ*_1_ is small enough. Figure 6 illustrates the basic idea.

The particles are resampled, and now the weighting is performed using the second factor, followed by resampling, etc. In this way the scheme slowly moves all particles towards the high‐probability regions of the posterior. Of course, after resampling several particles will be identical, so one needs to jitter the particles, so perturbing them slightly, to regain diversity.

This jittering should be a move of the particles that preserves the posterior pdf. It could be implemented as a MCMC method with the posterior as the target density (e.g. exploring resample‐move strategies; Doucet *et al.* 2001). However, a problem is that in sequential filtering we only have a representation of the posterior density in terms of the present particles, and this representation is very poor due to the small number of particles. Possible avenues are to fit a pdf of a certain shape to the present particles, e.g. a Gaussian mixture model, and use that as target density.

A problem in the geosciences is that this posterior fit needs to preserve the delicate balances between the model variables that are present in each particle, and an extra complication is that these balances can even be nonlinear. Also the transition kernel of the Markov chain should somehow preserve these balances. An example of its use in the geosciences is the Multiple Data Assimilations (MDA) method of Emerick and Reynolds (2013), in which the intermediate pdfs are assumed to be Gaussian. Evensen (2018) also gives a comparison of this method to other iterative implementations of the Ensemble Kalman Filter/Smoother.

However, if one allows for model error in the model equations, the following scheme proposed by Beskos *et al.* (2014) does not have this problem. In that case the prior at observation time can be written as (Equation 9)

(63) $$p(x^{n})\approx \frac{1}{N}\overset{N}{\underset{i=1}{\sum }}p(x^{n}|x_{i}^{n-1}),$$

in which we assume equal‐weight particles at time *n* − 1 for ease of presentation. In this case the MCMC method that has the posterior as invariant density is easy to find as the transition densities defined above, followed by an accept/reject step.

When several model time steps are performed between observation times, one can also perform tempering in the time domain, as explored in van Leeuwen (2003) and van Leeuwen (2009) in the Guided Particle Filter. The idea is to assimilate the observations ahead of time, with using as likelihood *p*(**y** ^∗^|**x** ^*m*^)^*γ*^), in which **y** ^∗^ is taken equal to the value **y** ^*n*^, and *γ* < <1. Here *m* < *n* is the present time of the model. This is then followed by a resampling step. The procedure can be followed over several time instances during the forward integration of the particles, increasing *γ*_*i*_ each time. At the observation time, *γ* = 1 is used. This will force the particles towards the observations and does not need extra jittering because each particle will see a different model noise realization ***β*** in the model integration after the resampling steps.

Of course one has to compensate for the fact that the transition density has been changed, and the way to do that is to realise that we have used importance sampling. Instead of sampling from $p(x^{m}|x_{i}^{m-1})$, we sample from a pdf $q(x^{m}|x_{i}^{m-1},$ $y^{n})\propto p(x^{m}|x_{i}^{m-1})p{\left(y^{n}|x^{m}\right)}^{\gamma }$, in which **y** ^∗^ is equal to **y** ^*n*^ taken at time *m*, and with larger observation uncertainty related to *γ*. This means that we have to compensate for the weights created by this sampling, so we need to introduce particle weights $w_{i}^{m}=p(x_{i}^{m}|x_{i}^{m-1})/q(x_{i}^{m}|x_{i}^{m-1},y^{*})\propto 1/p{\left(y^{*}|x_{i}^{m}\right)}^{\gamma }$ at each model time step we use this scheme.

The scheme generates extra weights during the model integration, but corrects for them at each new time when we resample, ensuring much better positioned particles at the actual observation time *n*. It has been used in a reduced‐gravity primitive equation model in van Leeuwen (2003), but not in high‐dimensional settings.

### 3.3 Particle flow filters

There is a recent surge in methods that dynamically move the particles in state space from equal‐weight particles representing the prior, *p*(**x**), to equal‐weight particles representing the posterior, *p*(**x**|**y**). In other words, one seeks a differential equation

(64) $$\frac{d}{ds}x=f_{s}(x)$$

in artificial time *s* ≥ 0 with the flow map defining the desired transformation. If the initial conditions of the differential Equation 64 are chosen from a pdf *p*_0_(**x**), then the solutions follow a distribution characterized by the Liouville equation

(65) $$\partial_{s}p_{s}=-\nabla_{x}\cdot (p_{s}f_{s})\;,$$

with initial condition *p*_0_(**x**) = *p*(**x**) and final condition $p_{s_{\text{final}}}(x)=p(x|y)$.

Two classes of particle flow filters arise. In the first we start from the tempering approach, such that *s*_final_ = 1. We now take the limit of more and more tempering steps by choosing *γ*_*i*_ = 1/*n* = Δ*s* with $\lim_{n\to \infty }$, so $\lim_{\gamma_{i}\to 0}$, or $\lim_{\Delta s\to 0}$ (Daum and Huang 2011, 2013, Reich 2011). This leads to

(66) $$\begin{array}{lll} & & \underset{\Delta s\to 0}{\lim}p_{s+\Delta s}(x)=p_{s}(x){\left(\frac{p(y|x)}{p(y)}\right)}^{\Delta s}\\ & & \;=p_{s}(x)\exp\left[\Delta s\left(\log p(y|x)-\log p(y)\right)\right]\\ & & \;\approx p_{s}(x)\left[1-\Delta s\log p(y|x)-\Delta s\log p(y)\right]. \end{array}$$

Hence we find

(67) $$\partial_{s}p_{s}(x)=-\nabla_{x}\cdot (p_{s}f_{s})=p_{s}(x)(\log p(y|x)-c_{s}),$$

with $c_{s}=\int p_{s}(x)\log p(y|x)dx$. Explicit expressions for *f*_*s*_ are available for certain pdfs such as Gaussians and Gaussian mixtures (Reich, 2012). These particle flow filters can be viewed as a continuous limit of the tempering methods described in the previous subsection, avoiding the need for resampling and jittering. Note that the elliptic partial differential equation 67 does not determine *f*_*s*_ uniquely. Optimal choices in the sense of minimizing the *L*_2_(*p*_*s*_)–norm of *f*_*s*_ lead to the theory of optimal transportation (Villani 2008; Reich and Cotter 2015).

**Figure 7:**
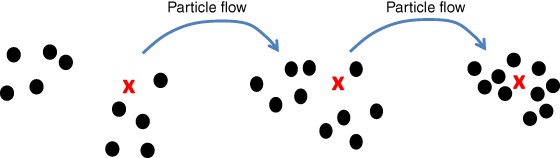
A typical particle flow filter. Left: the prior particles (dots), with one observation denoted by the red cross. Middle: the particles have moved over several artificial time steps towards the posterior. Note that the weights do not change. Right: the posterior particles after convergence of the filter, sampling the posterior directly [Colour figure can be viewed at wileyonlinelibrary.com]

Figure 7 shows the basic idea behind particle flow filters.

Alternatively, one can explore ideas from MCMC. One MCMC method that generates samples from the posterior is the Langevin Monte‐Carlo sampling, in which a sequence of samples is generated by

(68) $$x^{j+1}=x^{j}-\Delta s\nabla_{x}\log p(x|y)+\sqrt{2\Delta s}\beta^{j},$$

in which *β* ^*j*^ a random forcing term drawn from *N*(0,**I**). One can show that in the limit of *j*→*∞* these samples will be samples from the posterior. The corresponding Fokker–Planck equation for this stochastic PDE reads (e.g. Reich and Cotter 2015)

$$\begin{array}{ll} \partial_{s}p_{s} & =\nabla_{x}\cdot \left[p_{s}\nabla_{x}\{-\log p(x|y)\}\right]+\nabla_{x}\cdot \nabla_{x}p_{s}\;\\ & =-\nabla_{x}\cdot \left[p_{s}\left\{\nabla_{x}.\log p(x|y)-\nabla_{x}\log p_{s}\right\}\right].\; \end{array}$$

This equation corresponds to the deterministic PDE (64) in which **f**_*s*_(**x**) is given by:

(69) $$f_{s}(x):=\nabla_{x}\log p(x|y)-\nabla_{x}\log p_{s}(x)=-\nabla_{x}\log\frac{p_{s}(x)}{p(x|y)}.$$

Many other choices are possible that use

(70) $$\underset{s\to \infty }{\lim}p_{s}=p(x|y)$$

in Equation 65. An alternative approach, called Stein variational descent, has recently been proposed by Liu and Wang (2016). Stein variational descent can be viewed as a numerical approximation to a particle flow (64) with vector field

(71) $$f_{s}(x):=p_{s}\left\{\nabla_{x}\log p(x|by)-\nabla_{x}\log p_{s}(x)\right\}.$$

(Lu *et al.* 2019). We come back to this method below.

In general, to use any of these methods we need to be able to evaluate *p*_*s*_(**x**_*i*_), which is typically unknown as we only know the particle representation of *p*_*s*_(**x**). One way to solve this issue is to explore kernel embedding. A numerical implementation of the two formulations (69) and (71) can be based on a reproducing‐kernel Hilbert space (RKHS) F with reproducing kernel *K*(.,.), typically taken as a Gaussian. In the following, we will therefore assume that the kernel is symmetric *K*(**x**,**z**) = *K*(**z**,**x**). The inner product ${\left\langle g,f\right\rangle }_{F}$ in F satisfies the reproducing property

(72) $$g(x)={\left\langle K(x,\cdot ),g\right\rangle }_{F}\;.$$

A computational approximation to Equation 69 can now be obtained as follows (Degond and Mustieles, 1990; Russo, 1990). One approximates the pdf *p*_*s*_ by

(73) $$p_{s}(x)=\frac{1}{N}\overset{N}{\underset{j=1}{\sum }}K(x_{j},x)\;,$$

the vector field **f**_*s*_ by

(74) $$f_{s}(x)=\frac{\overset{N}{\underset{j=1}{\sum }}K(x_{j},x)u_{s}^{j}}{p_{s}(x)}\;,$$

and the *N* particles **x**_*j*_ move under the differential equations

(75) $$\frac{d}{ds}x_{j}=u_{s}^{j}\;.$$

Since the drift term Equation 69 gives rise to a gradient flow in the space of pdfs with respect to the Kullback–Leibler divergence KL = KL{*p*_*s*_||*p*(·|**y**)} between *p*_*s*_ and the posterior pdf (Reich and Cotter, 2015), it is natural to introduce the following particle approximation of the Kullback–Leibler divergence:

(76) $$V(\{x_{l}\}):={\left\langle p_{s},\log\frac{p_{s}}{p(\cdot |y)}\right\rangle }_{F}\;.$$

in the RKHS F and to set

(77) $$u_{s}^{j}:=-N\nabla_{x_{j}}V(\{x_{l}\})$$

in Equation 75, which leads to a gradient flow in the particles {**x**_*l*_} minimizing V. Details on the numerical implementation of this approach can be found in Pathiraja and Reich (2019).

The above formulation restricts the pdf *p*_*s*_, and hence the prior and the posterior, to be of the form Equation 73. Alternatively, one can embed the vector field of the flow in an appropriate reproducing kernel Hilbert space and not the density itself. With that we can derive a practical implementation of the Stein variational formulation (71) as follows. First, note that the change in KL due to the flow field **f**_*s*_ can easily be found as:

(78) $$\begin{array}{lll} dKL & = & \underset{\epsilon \to 0}{\lim}\frac{\text{KL}(p_{s+\epsilon })-\text{KL}(p_{s})}{\epsilon }\\ & = & -\int p_{s}(x)\left[f_{s}{\left(x\right)}^{T}\nabla_{x}\log p(x|y)+\nabla_{x}\cdot f_{s}(x)\right]dx.\\ & = & {\left\langle \nabla \text{KL},f_{s}\right\rangle }_{F}. \end{array}$$

where ∇KL is the gradient of KL, the maximal functional derivative of KL at every state vector **x** in the RKHS. Note that F here is different from the Hilbert space used earlier. Maximizing this change in KL as function of the flow field **f**_*s*_ is not trivial in general. However, with the reproducing kernel property of **f**_*s*_, we have

(79) $$f_{s}(x)=\left\langle K(\cdot ,x),f_{s}(\cdot )\right\rangle ,$$

in which K is a vector‐valued kernel, typically taken as K=IK. Using this in Equation 78, the gradient of the KL divergence is found as

(80) $$\nabla \text{KL}(x)=-\int p_{s}(z)\left[K(z,x)\nabla_{z}\log p(z|y)+\nabla_{z}K(z,x)\right]dz\;.$$

The important point is that this gradient is independent from **f**_*s*_. One now chooses **f**_*s*_ along this direction, which gives the steepest descent, as

(81) $$f_{s}(x)=-\epsilon \nabla \text{KL}(x).$$

Finally, one replaces the integral in Equation 80 by its empirical approximation, to obtain

(82) $$f_{s}(x_{j})=\epsilon \frac{1}{N}\overset{N}{\underset{l=1}{\sum }}\left[K(x_{l},x_{j})\nabla_{x}\log p(x_{l}|y)+\nabla_{x}K(x_{l},x_{j})\right]$$

for the dynamics (64) of the *N* particles **x**_*j*_.

The intuition behind Stein variational descent is that the first term in Equation 82 pulls the particles towards the mode of the posterior, while the second term acts as a repulsive force that allows for particle diversity. Liu and Wang (2016) derived this formulation for a steady‐state problem, and Pulido and van Leeuwen (2018) have extended the method to sequential particle filters. The scheme is given in Algorithm 9.


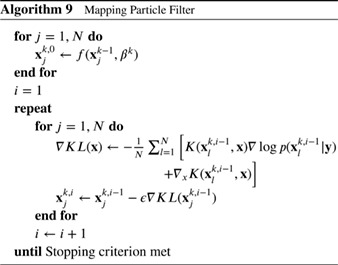


The free parameter of these methods is the reproducing kernel *K*(.,.), which needs to be chosen such that the particles sample the posterior and that physical (and potentially other) balances are retained. One also needs to select a proper time‐stepping scheme, typically chosen as a forward Euler scheme with variable time step *ϵ*, which can now be viewed as the step length in a gradient descent optimization algorithm.

### 3.4 Discussion

Viewing particle filters as a transportation problem from equal‐weight particles of the prior to equal‐weight particles of the posterior has led to an interesting set of filters. None of them have been implemented yet in high‐dimensional settings, but some of them are ready to do so. The strong involvement of the machine learning community in problems of this kind also suggests rapid progress here. Finally we mention that the equal‐weight particle filters from Section 2 can be viewed as one‐step transportation filters that explore the proposal density freedom, and in fact transform equal‐weight prior particles at time *n* − 1 to equal‐weight posterior particles at observation time *n*.

## 4 LOCALIZATION IN PARTICLE FILTERS

Localization is a standard technique in Ensemble Kalman filtering to increase the rank of the ensemble perturbation matrix, allowing for more observations to be assimilated, and to suppress spurious correlations where real correlations are very small, but ensemble correlations are larger because of sampling noise. Localization limits the influence of each observation to a localization area that is much smaller than the full model domain. This idea can easily be incorporated when calculating the particle weights locally, as pioneered by Bengtsson *et al.* (2003) and van Leeuwen (2003), and used in a high‐dimensional parameter estimation problem in Vossepoel and van Leeuwen (2006). The difficulty, as we shall see, lies in the resampling step: how does one generate 'smooth' global particles from locally resampled particles? Smooth is not well‐defined here, but it is related to the particles having realistic physical relations (balances) between the model variables. For example, if geostrophic balance is dominant, the resampling procedure should not generate particles that are completely out of geostrophic balance as that would lead to spurious adjustment processes via spurious gravity waves. Up to now localization is mainly used in connection with the standard particle filter, while more advanced proposals, apart from the optimal proposal, have not been explored. Farchi and Bocquet (2018) provide an excellent review of localization in particle filtering, treating a subset of the methods presented here, but including interesting extensions of the methods they describe.

The formal way localization can be introduced in particle filtering is as follows. Let us denote the state at grid point *k* as **x** ^*k*^. Hence, in contrast to other sections, a superscript here denotes not the time index, but the grid point. Note that in geoscience applications each grid point typically has several model variables, so **x** ^*k*^ is a vector in general. Physically it makes sense to assume that the posterior of the state at this grid point depends only on a subset of the observations. Let us denote that subset as **y** ^[*k*]^. We can then write

(83) $$p(x^{k}|y)\approx p(x^{k}|y^{\left[k\right]}).$$

In turn, these observations do not depend on the whole state vector but only on part of it, denoted by **x** ^(*k*)^:

(84) $$p(y^{\left[k\right]}|x)=p(y^{\left[k\right]}|x^{\left(k\right)}).$$

Introduce the notation **x** ^(*k*)∖*k*^ to denote all those grid points in that part of the state vector excluding grid point *k*. Then we can rewrite the above as an integral over the joint pdf:

(85) $$p(x^{k}|y^{\left[k\right]})=\int p(x^{\left(k\right)}|y^{\left[k\right]})\;dx^{(k)\setminus k}.$$

Exploring Bayes' theorem we find

(86) $$\begin{array}{lll} p(x^{\left(k\right)}|y^{\left[k\right]}) & = & \frac{p(y^{\left[k\right]}|x^{\left(k\right)})}{p(y^{\left[k\right]})}p(x^{\left(k\right)})\\ & \approx & \frac{1}{N}\overset{N}{\underset{i}{\sum }}\frac{p(y^{\left[k\right]}|x_{i}^{\left(k\right)})}{p(y^{\left[k\right]})}\delta (x^{\left(k\right)}-x_{i}^{\left(k\right)})\\ & = & \overset{N}{\underset{i}{\sum }}w_{i}^{\left(k\right)}\delta (x^{\left(k\right)}-x_{i}^{\left(k\right)}). \end{array}$$

Taken together, this shows that

(87) $$p(x^{k}|y^{\left[k\right]})\approx \overset{N}{\underset{i}{\sum }}w_{i}^{\left(k\right)}\delta (x^{k}-x_{i}^{k}).$$

**Figure 8:**
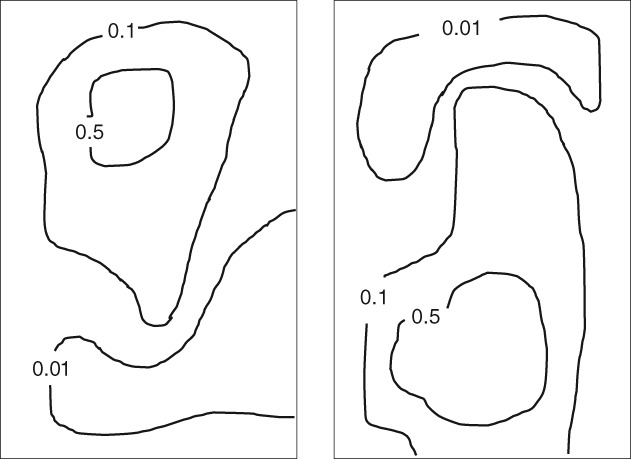
Illustration of a possible local weight distribution in a two‐dimensional domain, for two different particles. The particle on the left is close to observations in the central upper part of the domain, leading to high weights there, while the particle on the right is closer to observations in the central lower part of the domain, and hence higher weights there

The weights $w_{i}^{k}$ thus depend only on the local observations **y** ^[*k*]^ and the local prior particles $x_{i}^{\left(k\right)}$, so that the variance of the weights will be much smaller. Figure 8 illustrates how this local weighting could look for two different particles.

The approximation Equation 83 is not unrealistic: a temperature observation in New York is not expected to change our pdf of the temperature in London at the moment of the observation. Of course, there will be an effect at later times, but that is not relevant here. The same assumption underlies the use of localization in EnKFs, and in variational methods when the background‐error covariance is constructed.

However, mathematically it does not follow from the assumption that under the prior the values of the state at grid points separated by more than a certain distance are independent. There can be an indirect flow of information from observations far apart over observations between neigbouring grid points. In EnKFs, the Kalman gain is generally a dense matrix **HP** ^**f**^ **H**  + **R**, in which P^f^ is the forecast error covariance, is sparse, because its inverse (**HP** ^**f**^ **H**  + **R**)^−1^ can be dense. On the other hand, if **HP** ^**f**^ **H**  + **R** is diagonally dominant, then often its inverse is too.

Repeating the localization procedure for all grid points, we obtain all marginals of the posterior pdf. However, because the weights $w_{i}^{\left(k\right)}$ change from one grid point to the next, it is non‐trivial to obtain a consistent posterior for pairs of state values (**x** ^*k*^,**x** ^*ℓ*^) (and similarly for triplets, etc.). This can easily be seen using Figure 5: we would like to retain the left particle in the central upper half of the domain, and abandon elsewhere. That would mean that wherever it is abandoned, we need to replace it with another particle, perhaps partly with the particle in the right part of the figure. At the boundary between particles, a discontinuity will exist, which will lead to unphysical behaviour when this new particle is propagated forward in time.

This means that, to obtain global particles that can be forwarded with the model equations, one would need to somehow smoothly glue different particles together. This is a major problem and has hampered localization in particle filtering since the early 2000s. However, recently clever smoothing schemes have been constructed that seem to work well in high‐dimensional geophysical applications. We will report on those below.

Another issue is that the localization area cannot be too large to avoid filter collapse. As a rule of thumb, when there are more than (say) ten independent observations inside a local area, the particle filter will still tend to be degenerate for the number of O(10–1,000) particles one can typically afford. This means that, when the observation density is high, the localization areas have to become unphysically small, or observations have to be discarded. This issue might be solved using tempering techniques as discussed earlier, but is often avoided by artificially enforcing a minimal weight of the particles, or by changing the observations, for instance by projecting them on a lower‐dimensional space favoured by the prior.

Setting a minimal weight or projecting observations to a lower‐dimensional space favoured by the prior has a consequence that not all information will be extracted from the observations, as observations that are very different from the existing particles will be largely ignored. This is not directly equivalent to the standard quality‐control measures used by operational weather forecasting centres, in which observations that are a few standard deviations away from the forecast are ignored. The issue here is that a distance of less then one standard deviation for a few observations can already lead to weight collapse, and artificially setting minimum values for the weights avoids that.

### 4.1 Localization based on resampling

Several localization schemes have been proposed and discussed in the review by van Leeuwen (2009) and those will not be repeated here. The most obvious thing to do is to weight and resample locally, and somehow glue the resampled particles together via averaging at the edges between resampled local particles (van Leeuwen, 2003). In the following, several schemes in this category are discussed.

#### 4.1.1 Localized Particle Filter

Recently, Penny and Miyoshi (2016) used this idea with more extensive averaging, and their scheme runs as follows. First, for each grid point *j*, the observations close to that grid point are found and the weight of each particle *i* is calculated based on the likelihood of only those observations:

(88) $$w_{i,j}=\frac{p(y_{j}|x_{i,j})}{\overset{N}{\underset{k=1}{\sum }}p(y_{j}|x_{k,j})},$$

in which **y**_*j*_ denotes the set of observations within the localization area. Note the change of notation from the previous section, related to the explicit use of the particle index in all the following. This is followed by resampling via Stochastic Universal Resampling to provide ensemble members $x_{i,j}^{a}$ with *i* = 1,…,*N* for each grid point *j*.

Farchi and Bocquet (2018) extended this methodology by updating blocks of grid points locally, and introduce a smoothing operator in the weights (similar to Poterjoy 2016) as

(89) $$w_{i,j}=\frac{\overset{N_{j}}{\underset{k=1}{\sum }}G(d_{j,k}/h)(p(y_{k}|x_{i,k})}{\overset{N}{\underset{m=1}{\sum }}\overset{N_{j}}{\underset{k=1}{\sum }}G(d_{j,k}/h)p(y_{k}|x_{m,k})},$$

in which *G*(..) is a distance weighting function, e.g. a Gaussian or an approximation of that, *d*_*j*,*k*_ is the distance between grid points *j* and *k*, for each observation **y**_*k*_ at grid point *k* in the neighbourhood of grid point *j*. The parameter *h* is a distance radius, another tuning parameter. This formulation can be used for each grid point *j*, but also for each block of grid points *j*. They note that *G* can also be a Gaussian of a Gaussian, such that it works directly on $-\log p(y_{k}|x_{i,k})$.

As mentioned before, the issue is that two neighbouring grid points can have different sets of particles, and smoothing is needed to ensure that the posterior ensemble consists of smooth particles. This smoothing is performed by Penny and Miyoshi (2016) for each grid point *j* for each particle *i* by averaging over the *Np* neighbouring points within the localization area around grid point *j*:

(90) $$x_{i,j}^{a}=\frac{1}{2}x_{i,j}^{a}+\frac{1}{2Np}\overset{N_{p}}{\underset{k=1}{\sum }}x_{i,j_{k}}^{a},$$

in which *j*_*k*_ for *k* = 1,…,*N*_*p*_ denotes the grid point index for those points in the localization area around grid point *j*. The resampling via Stochastic Universal Resampling is done such that the weights are sorted before the resampling, so that high‐weight particles are joined up to reduce spurious gradients.

Farchi and Bocquet (2018) also suggest to smooth this operation as follows:

(91) $$x_{i,j}^{a}=\alpha x_{i,j}^{a}+(1-\alpha )\overset{N_{p}}{\underset{k=1}{\sum }}G(d_{j,j_{k}}/h)x_{i,j_{k}}^{a},$$

with *α* a tuning parameter. Note that by choosing *α* = 1/2 and $G(d_{j,j_{k}}/h)=1/N_{p}$, we recover the scheme by Penny and Miyoshi (2016).

While these schemes have been shown to solve the degeneracy problem in intermediate dimensional systems with fixed balances, like the barotropic vorticity model, it is unclear how they will perform in complex systems such as the atmosphere in which fronts can easily be smoothed out, and nonlinear balances broken, e.g. discussion in van Leeuwen (2009).

#### 4.1.2 Local Particle Filter

A different scheme that involves a very careful process of ensuring smooth posterior particles and retaining nonlinear relations has recently been proposed by Poterjoy (2016). An important difference with the state‐space localization methods discussed above is that observations are assimilated sequentially to avoid the discontinuity issues of the state‐space localization. This makes the algorithm non‐parallel, so slower than the state‐space localization methods, but Farchi and Bocquet (2018) demonstrate that a lower root‐mean square error (RMSE) can be achieved.

The scheme proceeds as follows. First, adapted weights are calculated for the first element *y*_1_ of the observation vector, as

(92) $${\tilde{w}}_{i}=\alpha p(y_{1}|x_{i})+1-\alpha .$$

These weights are then normalized by their sum $\tilde{W}$. Then the ensemble is resampled according to these normalized weights to form particles $x_{k_{i}}$.

The scalar *α* is an important parameter is this scheme, with *α* = 1 leading to standard weighting, and *α* = 0 leading to all weights being equal to 1 (before normalization). Its importance lies in the fact that the weights are always larger than 1 − *α*, so even a value close to 1, say *α* = 0.99, leads to a minimum weight of 0.01 that might seem small, but it means that particles that are more then 1.7 observational standard deviations away from the observations have their weights cut off to a value close to 1 − *α*. This limits the influence the observation can have on the ensemble. Furthermore, the influence of *α* does depend on the size of the observational error, which is perhaps not what one would like. It is included to avoid loosing any particle.

Now the following is done for each grid point *j*. For each member *i*, a weight is calculated as

(93) $${\tilde{\omega }}_{i}=\alpha \rho (1,j,r)p(y_{1}|x_{i})+1-\alpha \rho (1,j,r),$$

in which *ρ*(..) is the localization function with localization radius *r*. These weights are normalized with their sum over the particles, so a normalized weight *ω*_*i*_ for this grid point is obtained. Note, again, the role played by *α*. Then the posterior mean for this observation at this grid point is calculated as

(94) $${\bar{x}}_{j}=\overset{N}{\underset{i=1}{\sum }}\omega_{i}x_{i,j},$$

in which **x**_*i*,*j*_ is the state at grid point *j* of particle *i*. Next a number of scalars are calculated that ensure smooth posterior fields (Poterjoy, 2016) as detailed in Algorithm 10.

The final estimate becomes:

(95) $$x_{i,j}^{a}={\bar{x}}_{j}+r_{1j}(x_{k_{i},j}-{\bar{x}}_{j})+r_{2j}(x_{i,j}-{\bar{x}}_{j}),$$

where *k*_*i*_ is the index of the *i*'s sampled particle. This procedure is followed for each grid point so that at the end an updated set of particles is obtained that have incorporated the first observation. As a next step the whole process is repeated for the next observation, with the small change that ${\tilde{\omega }}_{i}$ is multiplied by ${\tilde{\omega }}_{i}$ from the previous observation, until all observations have been assimilated. In this way, the full weight of all observations is accumulated in the algorithm. Now the importance of *α* comes to full light: without *α* the ensemble would collapse because the $\tilde{\omega }$s would be degenerate when observations are accumulated.

The final estimate shows that each particle at grid point *j* is the posterior mean at that point plus a contribution from the deviation of the posterior resampled particle from that mean and a contribution from the deviation of the prior particle from that mean. So each particle is a mixture of posterior and prior particles, and departures from the prior are suppressed. When *α* = 1, so for a full particle filter, we find for grid points at the observation location, for which *ρ*(1,*j*,*r*) = 1, that *c*_*j*_ = 0, so *r*_2*j*_ = 0, and *r*_1*j*_≈1, so indeed the scheme gives back the full particle filter. The basic elements of the scheme are depicted in Algorithm 10.


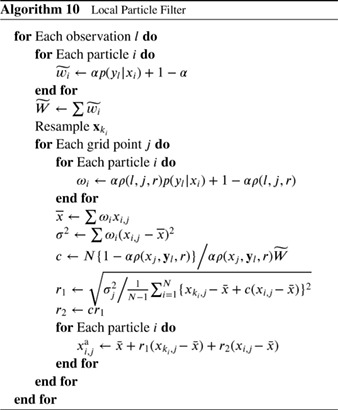


At grid points between observations, it can be shown that the particles have the correct first‐ and second‐order moments, but higher‐order moments are not conserved. Farchi and Bocquet (2018) generate a scheme that is quite similar, but they ensure correct first and second moments by exploring the localized covariances between observed and unobserved grid points directly in a regression step.) To remedy this, a probabilistic correction is applied at each grid point, as follows. The prior particles are dressed by Gaussians with width 1 and weighted by the likelihood weights to generate the correct posterior pdf. The posterior particles are dressed in the same way, each with weight 1/*N*. Then, the cumulative density functions (cdfs) for the two densities are calculated using a trapezoidal rule integration. A cubic spline is used to find the prior cdf values at each prior particle *i*, denoted by *cdf*_*i*_. Then a cubic spline is fitted to the other cdf, and the posterior particle *i* is found as the inverse of its cdf at value *cdf*_*i*_. Poterjoy (2016) gives details. The result of this procedure is that higher‐order moments are brought back into the ensemble between observation points.

This scheme, although rather complicated, is one of the two local particle filter schemes that has been applied to a high‐dimensional geophysical system based on primitive equations in Poterjoy and Anderson (2016). The other is the Localized Adaptive Particle Filter (LAPF) discussed below. (van Leeuwen 2003 applied a local particle filter to a high‐dimensional quasi‐geostrophic system, but that system is quite robust to sharp gradients as it does not allow for gravity waves.)

#### 4.1.3 Localized Adaptive Particle Filter

The LAPF is based on the localized version of the ensemble transform Equation 60 following the LETKF described in Hunt *et al.* (2007) and Reich (2013), with localization in observation space, and resampling in the spirit of Gaussian Mixture filters (Stordal *et al.* 2011). Localization is carried out around each grid point, and a transform matrix **D** is calculated for each localization box. We note that, as for the LETKF, the weights given by Equation 7 depend continuously on the box location and the observations.

In a first step, the observations are projected into the space spanned by the prior particles. As mentioned above, this will reduce the information extracted from the observations, but is perhaps less *adhoc* than setting a lower bound on the weights, as for instance used in the LPF. The LAPF carries out local resampling using universal resampling (e.g. van Leeuwen 2009).

In a second step, a careful adaptive sampling is carried out in ensemble space around each of the *N* temporary particles. This scheme runs as follows:

(a) Resampling is carried out based on a (radial) basis function centred at each particle. A simple case would be a Gaussian mixture, where the covariance of each of the centred Gaussians is taken as a scaled version *c* **P** of the local dynamical ensemble covariance **P**.

(b) The scaling factor *c* is individually calculated for each box based on the local observation‐ minus background‐error statistics. For details we refer to Potthast *et al.* (2019). By this, the LAPF guarantees to obtain a spread of the analysis ensemble which is consistent with the local dynamical observation minus background (o–b) statistics and the observation‐error covariance **R**. Further standard tools from the LETKF literature to control ensemble spread can be employed if needed.

(c) To obtain sufficient smoothness of the fields in physical space, the LAPF uses *N* global random draws to generate the resampling vectors around each particle in the space of ensemble coefficients. In combination with the fact that the LAPF draws in each box around each particle only – in a globally uniform way modulated by the ensemble covariance **P** and the factor *c* only – consistency and balance of the fields is achieved with sufficient precision. The scheme is depicted in Algorithm 11.


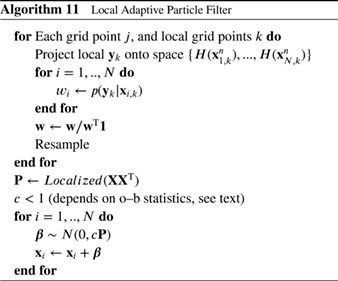


The LAPF is the first particle filter that has been implemented and tested in an operational numerical weather prediction context, and we provide a short description of the procedure. The method has been implemented in the DACE (Data Assimilation Coding Environment) system of Deutscher Wetterdienst (DWD; Potthast *et al.* 2019). The DACE environment includes a Local Ensemble Transform Kalman Filter (LETKF) based on Hunt *et al.* (2007), both for the global ICON model system and the convection‐permitting COSMO model system of DWD (Schraff *et al.* 2016), both of which are run operationally at DWD^1^ and build a basis, framework and reference for the LAPF particle filter implementation.

The ensemble data assimilation system is equipped with a variety of tools to control the spread of the ensemble, such as *multiplicative inflation* and *additive inflation*, *relaxation to prior spread* (RTPS), *relaxation to prior perturbations* (RTPP) and stochastic schemes to add spread to soil moisture and sea surface temperature (SST) when needed (details in Schraff *et al.* 2016).

Tests with the LAPF for the global ICON model with 40 particles of 40 km global resolution have been successfully and stably run over a duration of one month. Extensive tests on how many particles form the basis for resampling in each localization box have been carried out; the numbers vary strongly over the globe and all heights of the atmosphere, ranging from 1 to *N*, with relatively flat distribution. Diagnostics and tuning of the system is under development and is discussed in Potthast *et al.* (2019). Results show that the quality of the LAPF does not yet reach the scores of the operational global LETKF‐EnVAR system, but the system runs stably and forecast scores are about 10–15% behind the current operational system.

### 4.2 Local Ensemble Transform Particle Filter

This filter uses a classic sequential importance resampling particle filter from a set of forecast particles $x_{i}^{f}$, which can be obtained employing either the standard or the optimal proposals (or any other) and their associated importance weights $w_{i}^{f}$. The particles are then resampled in a statistically consistent manner, which can be characterized by an *N* × *N* stochastic transition matrix **D** with the following properties: all entries *d*_*ij*_ of **D** are non‐negative and

(96) $$\overset{N}{\underset{i=1}{\sum }}d_{ij}=1\;,\;\frac{1}{N}\overset{N}{\underset{j=1}{\sum }}d_{ij}=w_{i}^{f}\;.$$

Let us denote the set of all such matrices by D. Then any D∈D leads to a resampling scheme by randomly drawing an element *j* ^∗^ ∈ {1,…,*N*} according to the probability vector $p_{j}=(p_{1j},\text{…},p_{Nj})\in R^{N}$ for each *j* = 1,…,*N*. The *j*th forecast particle $x_{j}^{f}$ is then replaced by $x_{j^{*}}^{f}$ and the new particles $x_{j}^{n}=x_{j^{*}}^{f}$, *j* = 1,…,*N*, provide an equally weighted set of particles from the posterior distribution. Note that multinomial resampling corresponds to the simple choice

(97) $$d_{ij}=w_{i}^{f}.$$

The ensemble transform particle filter (ETPF; Reich 2013, Reich and Cotter 2015) is based on the particular choice $\hat{D}\in D$ that minimizes the expected squared Euclidian distance between forecast particles, i.e.

(98) $$\hat{D}=\arg\underset{D\in D}{\min}\overset{N}{\underset{i,j=1}{\sum }}d_{ij}\|x_{i}^{f}-x_{j}^{f}{\|}^{2}\;.$$

It has been shown under appropriate conditions that the variance of a resampling step based on $\hat{D}$ vanishes as *N*→*∞* (McCann, 1995; Reich, 2013). This fact is utilized by the ETPF and one defines

(99) $$x_{j}^{n}=\overset{N}{\underset{i=1}{\sum }}x_{i}^{f}{\hat{d}}_{ij}$$

even for finite particles numbers. Of course, by its very construction, the ETPF underestimates the posterior covariance. However, there are corrections available that lead to second‐order accurate implementations (de Wiljes *et al.* 2017). Section 5.3 gives more details.

Following previously introduced notations, localization can now be implemented into the ETPF as follows. For each grid point *k*, we extract the values of the forecast particle $x_{i}^{f}$ at that grid point and denote them by $x_{i}^{k}$. Using the observations local to this grid point, we calculate localized importance weights $w_{i}^{k}$ for $x_{i}^{k}$. Then Equation 98 gives rise to a localized transformation matrix

(100) $${\hat{D}}^{k}=\arg\underset{D\in D^{k}}{\min}\overset{N}{\underset{i,j=1}{\sum }}d_{ij}\|x_{i}^{k}-x_{j}^{k}{\|}^{2}$$

at grid point *k* with the set $D^{k}$ defined by

(101) $$D^{k}=\left\{D\in R_{+}^{N\times N}:\overset{N}{\underset{i=1}{\sum }}d_{ij}=1,\;\overset{N}{\underset{j=1}{\sum }}d_{ij}=w_{i}^{k}N\right\}.$$

Note that the transport cost (distance) $t_{ij}=\|x_{i}^{k}-x_{j}^{k}{\|}^{2}$ can be replaced by any other localized cost function. Chen and Reich (2015) give more details. The transport problem (Equation 100) at each grid point can be computationally expensive. Less expensive approximations, such as the Sinkhorn approximation, and their implementation into the localized ETPF (LETPF) are discussed in de Wiljes *et al.* (2017). Farchi and Bocquet (2018) have extended this algorithm to block weighting, similar to their extension of the Local Particle Filter.

The latter authors also defined a local transform particle filter in state space. This involves a transformation, at each grid point, from prior to posterior particles by a transformation, which essentially becomes an anamorphosis step. The prior and posterior probability densities need to be known as continuous densities, and Farchi and Bocquet (2018) use kernel density estimation with the particles as basis. The interesting suggestion is that, since the transformation is deterministic and expected to be smooth over the space coordinates, no specific smoothing is needed after the transformation. We refer to their paper for details on this methodology.

### 4.3 Space–time particle filters

The idea to run a particle filter over the spatial domain was introduced by van Leeuwen (2009), and the first algorithm, the Location Bootstrap Filter, was published by Briggs *et al.* (2013). The Space–Time Particle Filter by Beskos *et al.* (2017) improves on this algorithm by removing the jitter step, as explained below. In the following we assume observations at every grid point, but the algorithms can easily be adapted to other observation networks.

The Location Particle Filter of Briggs *et al.* (2013) runs as follows. The grid points are ordered 1,…,*L*, such that points *l* and *l* + 1 are neighbouring grid points for each *l* ∈ 1,…,*L*. In each grid point *l* we have a sample **x**_*i*,*l*_ for *i* ∈ 1,…,*N*, and *l* denotes the grid point number. We start the spatial particle filter at location *l* = 1 by calculating the weight *p*(**y**_1_|**x**_*i*,1_) (where the time index is suppressed) for each prior particle *i*, and perform resampling using these weights over the whole spatial domain. This means that the resampled particles are now samples of *p*(**x** ^1:*L*^|**y** ^1^). A small amount of jitter is added to avoid identical particles. The choice of this jitter density is again not clear for geophysical applications; more research is needed on this issue.

Then, the algorithm moves to the next grid point, calculates the weights *p*(**y**_2_|**x**_*i*,2_), and resamples the full state particles using this weight, generating samples from *p*(**x**_1:*L*_|**y**_1_,**y**_2_). Again some jitter is needed to avoid ensemble collapse, and the algorithm moves to the next grid point, until all grid points are treated this way. Algorithm 12 describes the computational steps.


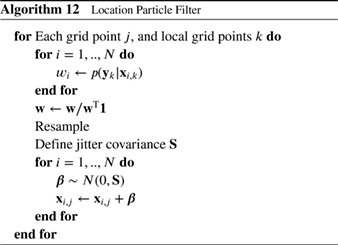


Note that the algorithm does not suffer from artificial sharp gradients because all resampled particles are global particles, but the algorithm will be very sensitive to the choice of the jitter density used after updating the ensemble in each grid point. Furthermore, when prior and posterior are very different, the algorithm will perform poorly, and Briggs *et al.* (2013) propose a smoother variant that employs copulas for numerical efficiency. We will not discuss that variant here.

Beskos *et al.* (2017) introduce the Space–Time Particle Filter. Instead of using a jitter density to avoid identical particles, they exploit the spatial transition density $p(x_{l}^{n}|x_{l-1}^{n,1},x_{1:L}^{n-1})$, in which *n* is the time index and *l* the spatial index. (In fact, Beskos *et al.* (2017) allow for a proposal density, but we will explain the algorithm using the prior spatial pdf as proposal.) So they exploit the pdf of the state at time *n* and grid point *l*, $x_{l}^{n}$, conditioned on all previous grid points $x_{1:l-1}^{n}$ at the same time *n*, and conditioned on all grid points at time *n* − 1, denoted $x_{1:L}^{n-1}$. They do this by introducing a set of *M* local particles *j*, for each global particle *i*, with *i* ∈ 1,…,*N*.

For each of the global particles *i* they run the following algorithm over the whole grid:

1. Starting from location *l* = 1, the *M* local particle filters grow in dimension when moving over the grid towards the final position *L*. At the first grid point, the prior particles at that grid point are used, weighted with the local likelihood *p*(**y**_1_|**x**_1_) and resampled. Let us call these particles ${\hat{x}}_{j,1}$, in which *j* is the index of the local particle, and 1 is the index of the grid point.
2. The mean ${\bar{w}}^{1}$ of the unnormalized weights is calculated.
3. For the next grid point, each of these *M* resampled particles is propagated to that grid point by drawing from $p(x_{2}|{\hat{x}}_{j,1},x_{j,1:L}^{n-1})$. Since each of the *M* particles is drawn independently, they will differ and no jittering is needed.
4. Then the unnormalized weights *p*(**y**_2_|**x**_2_) are calculated, and their mean ${\bar{w}}^{2}$, followed by a resampling step.
5. This process is repeated until *l* = *L*, so until the whole space is covered.
6. Finally, the total weight $w_{1}=\prod_{l=1}^{L}{\bar{w}}^{l}$ is calculated, which is the unnormalized weight of the first global particle.

Algorithm 13 summarizes the scheme.


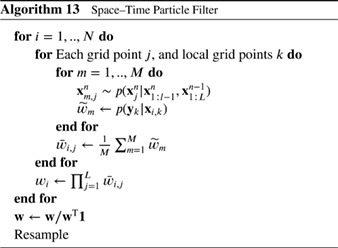


This procedure is followed *N* times for each global particle *i* independently. These global particles are then resampled according to the weight *G*_*i*_. It is still possible that this filter is degenerate; Beskos *et al.* (2017) gives details and potential solutions.

The importance of this filter lies in the fact that there is a formal proof that it converges to the correct posterior for an increasing number of particles, unlike any of the other algorithms discussed. Furthermore, the authors show that degeneracy can be avoided if the number of particles grows as the square of the dimension of the system – much faster convergence than e.g. the optimal proposal density.

### 4.4 Discussion

Following in the footsteps of EnKFs, exploring localization in particle filters is a rapidly growing field. But localization in particle filters is not trivial as there is no automatic smoothing via smoothed sample covariances as in EnKFs. Most local particle filters impose explicit spatial smoothing, which can affect delicate balances in the system. Worth mentioning in this context is the localization introduced by Robert and Künsch (2017), who process observations sequentially in their hybrid Ensemble Kalman Filter–Particle Filter approach such that the second‐order properties of the particle filter part remain correct. This method is discussed in the next chapter. The ETPF and the LAPF come closest to the EnKF by using a linear transportation matrix to transforms the prior ensemble into a posterior ensemble, and this matrix can be made smoothly varying with space. All of these smoothing operations rely on forming linear combinations of particles, so can potentially harm nonlinear balances in the model. Furthermore, it should be noted that the smoothing operation does not necessarily follow Bayes' theorem, so it might result in an extra approximation of the true posterior pdf. However, when the ensemble size is small, this approximation might be negligible compared to the Monte‐Carlo noise from the finite ensemble size.

The Location Particle Filter and the Space–Time Particle Filter avoid this smoothing and rely on statistical connections between different grid points. The former does this via the prior pdf, defined by the prior particles. When the number of particles is low, this pdf is estimated rather poorly. Furthermore, the method needs jittering of the global particles to avoid ensemble collapse after every resampling step after each new observation is assimilated. This jittering pdf can be chosen arbitrarily, for instance a smooth Gaussian, but it does violate Bayes' theorem. As mentioned above, this error might be negligible when the ensemble size is small. The latter method explores the transition density over space and time, leading to consistent estimates of the spatial relations between grid points. Another potential issue of both methods is that, if the spatial field is two‐ (or higher) dimensional, as in geoscience applications, it is unclear how to order the grid points, and potentially large jumps might be created between neighbouring grid points that are treated as far apart by the algorithm. This needs further investigation.

## 5 HYBRIDS BETWEEN PARTICLE FILTERS AND ENSEMBLE KALMAN FILTERS

As mentioned in the previous section, there are two issues with localization. Firstly, particle filters that employ resampling need to ensure smooth updates in space so that the newly formed global particles do not encounter strong adjustments to physical balances due to artificial gradients from glueing particles together. Present‐day localized particle schemes concentrate on this issue.

Secondly, the localization area cannot contain too many independent observations, and as a rule of thumb ten independent observations is often too many, to avoid weight collapse. As mentioned, this demand can be in strong contrast with physical considerations of appropriate length‐scales. This is one of the main reasons to consider hybrids between particle filters and EnKFs within a localization scheme. In the following, several recent hybrid methods are presented.

### 5.1 Adaptive Gaussian Mixture Filter

A bridging formulation allows to smoothly transition between an ensemble Kalman filter and a particle filter analysis update. One such formulation is the adaptive Gaussian mixture filter (Stordal *et al.* 2011).

In a Gaussian mixture filter, the distribution is approximated by a combination of normal distributions centred at the values of the particles. Thus we have

(102) $$p(x^{n})=\overset{N}{\underset{i=1}{\sum }}w_{i}N\left(x_{i}^{f},{\hat{P}}^{f}\right),$$

where $N(x_{i}^{f},{\hat{P}}^{f})$ is a Gaussian Kernel with mean $x_{i}^{n}$ and covariance ${\hat{P}}^{f}$. This covariance is initialized from the sample covariance matrix **P** ^f^ of the ensemble by multiplying with a so‐called bandwidth parameter 0 < *h* ≤ 1 such that

(103) $${\hat{P}}^{f}=h^{2}P^{f}.$$

At the analysis time, the filter computes a two‐step update: in the first step we update the ensemble members and the covariance matrix according to the Kalman filter equations given by

(104) $$X^{n}=X^{f}+{\hat{K}}^{n}\left(y^{n}1^{T}-HX^{f}\right),$$

(105) $${\hat{K}}^{n}={\hat{P}}^{f}H^{T}{\left(H{\hat{P}}^{f}H^{T}+R^{n}\right)}^{-1}$$

and

(106) $$P^{n}=\left(I-{\hat{K}}^{n}H\right){\hat{P}}^{f}.$$

Note that this is just a shorthand notation for updating each centre for the prior Gaussians. For computational efficiency, the analysis equations in the (adaptive) Gaussian mixture filter (Hoteit *et al.* 2008; Stordal *et al.* 2011) were proposed to use a factorized covariance matrix in the form ${\hat{P}}^{f}=LUL^{T}$, as can be obtained from a singular value decomposition of the ensemble perturbation matrix and used, for example, in the Singular Evolutive Interpolated Kalman (SEIK) filter (Pham, 2001) and Error‐Subspace Transform Kalman Filter (ESTKF; Nerger *et al.* 2012). However, the particular form of the Kalman filter update equations is not crucial here.

In the second step we update the weights of the particles according to

(107) $$w_{i}^{n}\approx w_{i}^{n-1}N_{y^{n}|x^{f}}\left(Hx_{i}^{f},R^{n}\right),$$

in which $R^{n}=R+H{\hat{P}}^{f}H^{T}$, and then normalize these so that the sum of the weights is one.

The bridging is now done by interpolating the analysis weight with a uniform weight *N* ^−1^ as

(108) $$w_{i}^{\left(\alpha \right)}=\alpha w_{i}+(1-\alpha )N^{-1},$$

where *α* is the bridging parameter. We obtain a transition between the EnKF and the particle filter by varying both *α* and *h*. For *α* = 0 and *h* = 1, we obtain the uniform weights of the EnKF, while for *α* = 1 and *h* = 0 we obtain the particle filter weights. Stordal *et al.* (2011) proposed to adaptively estimate an optimal value of *α* by setting $\alpha =N^{-1}{\hat{N}}_{\text{eff}}$ where ${\hat{N}}_{\text{eff}}={\left(\sum_{i}w_{i}^{2}\right)}^{-1}$ is the effective sample size.

The update formulation of the adaptive Gaussian mixture filter reduces the risk of ensemble degeneracy, but cannot fully avoid it. To this end, we can combine the filter with a resampling step as in other particle filters.

### 5.2 Ensemble Kalman Particle Filter

**Figure 9:**
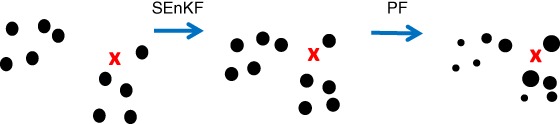
The Ensemble Kalman Particle Filter. First a Stochastic EnKF is performed, followed by a standard Particle Filter [Colour figure can be viewed at wileyonlinelibrary.com]

The Ensemble Kalman Particle Filter of Frei and Künsch (2013) is a hybrid EnKF‐PF. It is based on tempering in just two steps, splitting the likelihood into two factors

(109) $$p(x^{n}|y^{n})=p{\left(x^{n}|y^{n}\right)}^{\alpha }\;p{\left(x^{n}|y^{n}\right)}^{1-\alpha },$$

with *α* ∈ (0,1). In the first step the Stochastic Ensemble Kalman filter of Burgers *et al.* (1998) is applied, and in the second step a particle filter. When the parameter *α* is close to 0, the scheme is like a full particle filter, while for *α* close to 1 it is essentially the ensemble Kalman filter. Figure 9 illustrates the idea.

Two problems with a direct application of the above scheme are identified by Frei and Künsch (2013): the particle filter weights are influenced by the random modelled observations in the Stochastic EnKF (SEnKF), and the resampling step in the particle filter will lead to identical particles. To avoid both, the algorithm is modified as follows. Firstly, assuming a Gaussian likelihood, the SEnKF particles can be written as:

(110) $$x_{i}^{\text{SEnKF}}=x_{i}+K_{\alpha }(y-Hx_{i}-\epsilon_{i}),$$

with ***ϵ***_*i*_∼*N*(0,**R**/*α*) and **K**_*α*_ is the normal gain, but with **R** divided by *α*. Thus, the particles can be seen as draws from

(111) $$x_{i}^{\text{SEnKF}}∼N(\nu_{i},P^{\text{EnKF}})$$

in which

(112) $$\nu_{i}=x_{i}+K_{\alpha }(y-Hx_{i})$$

and

(113) $$P^{\text{SEnKF}}=\frac{1}{\alpha }K_{\alpha }RK_{\alpha }^{T}.$$

Hence the SEnKF posterior can be written as

(114) $$p{\left(x|y\right)}^{\text{SEnKF}}=\frac{1}{N}\overset{N}{\underset{i=1}{\sum }}N(\nu_{i},P^{\text{SEnKF}}).$$

Instead of performing the standard SEnKF sampling from this density, we delay that sampling and perform the multiplication with the second likelihood *p*(**y**|**x**)^1 − *α*^ analytically. This is easy because the EnKF posterior is a Gaussian mixture and the likelihood is a Gaussian, so the full posterior is a Gaussian mixture too. This leads to a full posterior

(115) $$\overset{N}{\underset{i=1}{\sum }}\gamma_{i}N(\mu_{i},P^{\text{PF}})$$

in which

(116) $$\mu_{i}=\nu_{i}+\hat{K}(y-H\nu_{i}),$$

(117) $$\gamma_{i}=N\left(y-H\nu_{i},HP^{\text{SEnKF}}H^{T}+R/(1-\alpha )\right),$$

(118) $$P^{\text{PF}}=(I-\hat{K}H)P^{\text{SEnKF}},$$

where

(119) $$\hat{K}=P^{\text{SEnKF}}H^{T}{\left(HP^{\text{SEnKF}}H^{T}+R/(1-\alpha )\right)}^{-1}.$$

Note that the normalization constants in *γ*_*i*_ do not have to be calculated as we know that they should fulfil $\sum_{i}\gamma_{i}=1$.

The way to sample the particles now becomes a two‐step procedure. First draw *N* samples from the distribution of the mixture coefficients *γ*_*i*_ and then draw from the selected Gaussian mixture components:

(120) $$x_{i}^{\text{EKPF}}=\mu_{k_{i}}+\xi_{i},$$

in which *k*_*i*_ denotes the resampled particle index *i* and $\xi_{i}∼N(0,P^{\text{PF}})$. The variables ***ξ***_*i*_ can again be generated in two steps by

(121) $$\xi_{i}=(I-\hat{K}H^{T})K_{\alpha }\epsilon_{i,1}+\hat{K}\epsilon_{i,2},$$

where ***ϵ***_1.*i*_ and ***ϵ***_*i*,2_ are independent draws from *N*(0,**R**/*α*) and *N*(0,**R**/(1 − *α*)), respectively.

The scheme is very closely related to a Gaussian mixture model, as the EnKF step forces the prior for the particle filter to be a Gaussian mixture. The strong point of this scheme is that the width of each Gaussian follows naturally from the stochastic part of the EnKF, while it is *adhoc* in standard Gaussian mixture models. Furthermore, while the standard Gaussian mixture model uses the observation covariance matrix **R**, this filter uses an inflated **HP** ^SEnKF^ **H** ^T^ + **R**/(1 − *α*), which will lead to a better weight distribution. Finally, the starting points of the centres of the prior Gaussians will be closer the observations, suggesting more uniform weights. The pseudocode of the scheme is presented in Algorithm 14.


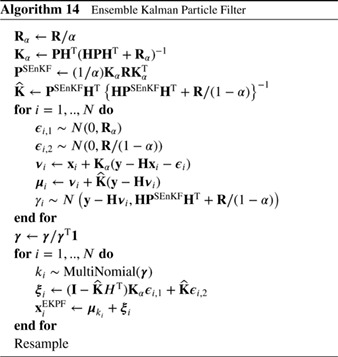


In an extension of the scheme, Frei and Künsch (2013) suggest forming a tempering scheme, alternatively using the EnKF and the particle filter. The resampling step of the particle filter is not problematic in this case as the Kalman filter will diversify identical particles in each next iteration. The paper also discusses approximate schemes for non‐Gaussian observation errors and nonlinear observation operators.

In Robert *et al.* (2018), a variant of this method has been introduced which is based on the LETKF instead of the stochastic variant and in which the update is in ensemble space:

(122) $$X^{\text{EKPF}}=X^{f}W,$$

where the column sums of **W** equal 1. The matrix **W** can be split into

(123) $$W=W^{\mu }W^{\alpha }+W^{\xi }$$

where **W** ^*μ*^ corresponds to computing the centres ***μ***_*i*_, **W** ^*α*^ to the resampling and **W** ^*ξ*^ to the added noise ***ξ***_*i*_. In the transform variant, **W** ^*ξ*^ is deterministic and chosen such that the sample covariance of **X** ^PI^ equals the covariance of the Gaussian mixture Equation 115. It thus belongs also to the class of second‐order exact filters discussed in the next section.

Robert *et al.* (2018) apply a localized transform Ensemble Kalman Particle Filter in the KENDA (Kilometer‐Scale Ensemble Data Assimilation) system with a set‐up similar to the one used operationally by MeteoSwiss. This system computes the weight matrices **W** only on a coarse grid and then interpolates these matrices to the original grid. Therefore the discontinuities introduced by resampling are smoothed out, but in a way that is possibly optimal for the EnKF and not for the EnKPF. In Robert and Künsch (2017) a different localization method for the EnKPF was developed which proceeds by sequentially assimilating observations *y* ^*k*^, limiting the state components influenced by *y* ^*k*^ to a subset. It smoothes out the discontinuities that occur when a resampled particle in the region influenced by *y* ^*k*^ is connected to a background particle outside of this region. The smoothing is done in such a way that the second‐order properties of the smoothed particle remain correct.

### 5.3 Second‐order exact filters

A second‐order exact filter ensures that the posterior ensemble mean and ensemble covariance matrix are equal to those obtained from the particle filter weights. Thus, the requirement for the mean of the analysis ensemble is

(124) $${\bar{x}}^{n}=\frac{1}{N}\overset{N}{\underset{i=1}{\sum }}x_{i}^{n}=\overset{N}{\underset{i=1}{\sum }}w_{i}x_{i}^{f},$$

where the superscript f denotes the forecasted state vector. Likewise, the posterior ensemble covariance matrix is required to fulfil

(125) $$P^{a}=\frac{1}{N}\overset{N}{\underset{i=1}{\sum }}\left(x_{i}^{n}-{\bar{x}}^{n}\right){\left(x_{i}^{n}-{\bar{x}}^{n}\right)}^{T}$$

(126) $$=\overset{N}{\underset{i=1}{\sum }}w_{i}\left(x_{i}^{f}-{\bar{x}}^{n}\right){\left(x_{i}^{f}-{\bar{x}}^{n}\right)}^{T}.$$

#### 5.3.1 Merging particle filter

The merging particle filter by Nakano *et al.* (2007) explores the sampling aspect of the resampling step. The method draws a set of *q* ensembles each of size *N* from the weighted prior ensemble at the resampling step. Then these sets are merged via a weighted average to obtain a new set of particles that has the correct mean and covariance but is more robust than the standard particle filter. Define **x**_*i*,*j*_ as ensemble member *i* in ensemble *j*. The new merged ensemble members are generated via

(127) $$x_{i}^{a}=\overset{q}{\underset{j=1}{\sum }}\alpha_{j}x_{i,j}.$$

To ensure that the new ensemble has the correct mean and covariance, the coefficients *α*_*j*_ have to be real and need to fulfil the two conditions

(128) $$\overset{q}{\underset{j=1}{\sum }}\alpha_{j}=1;\;\overset{q}{\underset{j=1}{\sum }}\alpha_{j}^{2}=1.$$

When *q* > 3, there is no unique solution for the *α*s, while for *q* = 3 one finds

(129) $$\alpha_{1}=\frac{3}{4};\;\alpha_{2}=\frac{\sqrt{13}+1}{8};\;\alpha_{3}=-\frac{\sqrt{13}-1}{8}.$$

We can make the weights space‐dependent in high‐dimensional systems and, since the new particles are merged previous particles, the resulting global particles are expected to be smooth. The scheme is depicted in Algorithm 15.


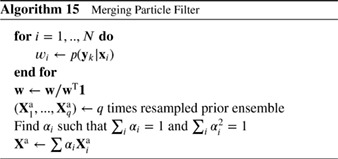


#### 5.3.2 Nonlinear Ensemble Transform Filter

A simple formulation of a second‐order exact filter can be obtained by using Equation 124 to compute the mean of the posterior ensemble (Xiong *et al.* 2006; Tödter and Ahrens, 2015). For the associated ensemble perturbations, we can derive from Equation 126 with **w** = (*w*_1_,…,*w*_*N*_)^T^ and **W** = diag(**w**) that

(130) $$P^{a}=X^{f}\left(W-ww^{T}\right){\left(X^{f}\right)}^{T}.$$

Posterior ensemble perturbations can now be obtained by factorizing **A** = **W** − **ww** ^T^, for example, by a singular value decomposition as **A** = **VΛ** *V* ^T^. This leads to **A** ^1/2^ = **VΛ** ^1/2^ **V** ^T^ and posterior perturbations are then given by

(131) $${X\prime }^{n}=\sqrt{N}X^{f}V\Lambda^{1/2}V^{T}.$$

Finally, the full posterior particles are given by

(132) $$x_{i}^{n}=X^{f}{\left(w1^{T}+\sqrt{N}V\Lambda^{1/2}V^{T}\right)}_{i}.$$

The computations of this filter are very similar to those in ensemble square‐root Kalman filters like the ETKF (Hunt *et al.* 2007) or ESTKF (Nerger *et al.* 2012). As such, we can can also localize the filter in the same way. The localized NETF has been successfully applied to a high‐dimensional geophysical system based on primitive equations in Tödter *et al.* (2016). In addition, the filter can be easily extended to a smoother by applying the filter transform matrix (the term in parentheses in Equation 132) to previous analysis times (Kirchgessner *et al.* 2017). The scheme is depicted in Algorithm 16.


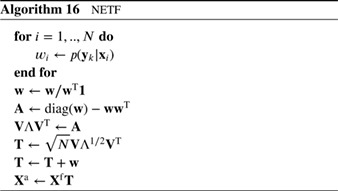


#### 5.3.3 Nonlinear ensemble adjustment filter

There is also a stochastic variant of the previous algorithm (Lei and Bickel, 2011), which is motivated from the Stochastic Ensemble Kalman filter (Burgers *et al.* 1998; Houtekamer and Mitchell, 1998). In this filter, we generate a set of perturbed model observations

(133) $$y_{i}=H(x_{i})+\epsilon_{i},\;\;\;i=1,\text{…},N,$$

which represents the observation probability distribution. We now obtain an analysis mean of each particle analogously to Equation 124 by

(134) $${\bar{x}}^{n}(y_{k})=\overset{N}{\underset{i=1}{\sum }}w_{i}(y_{k})x_{i}^{f},$$

where each weight *w*_*i*_(**y**_*k*_) is computed from the likelihood of the perturbed measured ensemble member **H**(**x**_*i*_) . When we now define

(135) $${\hat{P}}^{a}(y_{k})=\overset{N}{\underset{i=1}{\sum }}w_{i}(y_{k})\left(x_{i}^{f}-{\bar{x}}^{n}(y_{k})\right){\left(x_{i}^{f}-{\bar{x}}^{n}(y_{k})\right)}^{T},$$

we obtain the posterior ensemble members as

(136) $$x_{k}^{n}={\bar{x}}^{n}+{\left(P^{a}\right)}^{1/2}{\hat{P}}^{a}{\left(y_{k}\right)}^{-1/2}\{x_{k}^{f}-{\bar{x}}^{n}(y_{k})\},$$

where ${\bar{x}}^{n}$ is given by Equation 124 and *P* ^a^ is given by Equation 126. This update equation only yields the correct first and second moments of the posterior distribution in the limit of a large ensemble.

#### 5.3.4 Second‐order exact ETPF

The ETPF (Section 4.2) can also be formulated to be second‐order accurate (de Wiljes *et al.* 2017). For this, we approximate

(137) $$A=W-{\mathrm{ww}}^{T}\approx \frac{1}{N}\left(\hat{D}-{\mathrm{w1}}^{T}\right){\left(\hat{D}-{\mathrm{w1}}^{T}\right)}^{T},$$

where the matrix $\hat{D}$ is obtained through Equation 98. To ensure the second‐order accuracy, we introduce a correction term such that

(138) $$\tilde{D}=\hat{D}+\Delta ,$$

with **Δ** being a symmetric *N* × *N* matrix. Using $\tilde{D}$ in Equation 137 and requiring that the result is equal to **A** leads to the condition

(139) $$\begin{array}{ll} N(W-{\mathrm{ww}}^{T}) & -(\hat{D}-{\mathrm{W1}}^{T}){\left(\hat{D}-{\mathrm{W1}}^{T}\right)}^{T}\;\\ & =(\hat{D}-{\mathrm{W1}}^{T})\Delta +\Delta {\left(\hat{D}-{\mathrm{W1}}^{T}\right)}^{T}+\Delta \Delta ,\; \end{array}$$

which is a quadratic equation in **Δ** in the form of a continuous‐time algebraic Riccati equation and there are known solution methods for this type of equation (e.g. de Wiljes *et al.* 2017). Note that $\tilde{D}$ still satisfies Equation 96. However, ${\tilde{d}}_{ij}\geq 0$ no longer holds, in general.

### 5.4 Hybrid LETPF–LETKF

The hybrid LETPF–LETKF is also based on the simple idea of splitting the likelihood function into two factors at each grid point *k*, i.e.

(140) $$p(x^{k}|y^{\left(k\right)})=p{\left(x^{k}|y^{\left(k\right)}\right)}^{1-\alpha }\;p{\left(x^{k}|y^{\left(k\right)}\right)}^{\alpha },$$

with *α* ∈ (0,1), but now the particle filter is employed first, followed by the ensemble Kalman filter. This is similar to tempering in just two steps. When the likelihood is Gaussian, the posterior is expected to be more Gaussian than the prior. Hence it makes sense to use a particle filter in the first step, and to try to use an EnKF in the second step of the tempering procedure.

If the likelihood is Gaussian with localized error covariance matrix **R** ^*k*^, then the factorization is equivalent to scaling this matrix by 1/*α* and 1/(1 − *α*), respectively. Hence, one can, for example, first apply an LETPF to the forecast particles $x_{i}^{f}$ with inflated covariance matrix **R** ^*k*^/*α* in order to obtain new particle values

(141) $$\tilde{x_{i}^{k}}=\overset{N}{\underset{j=1}{\sum }}d_{ij}^{k}(\alpha )x_{i}^{k}$$

at each grid point *k*. One then applies the LETKF to these intermediate particles ${\tilde{x}}_{i}$ with inflated covariance matrix **R** ^*k*^/(1 − *α*). The choice of *α* is, of course, crucial. Numerical experiments indicate (Chustagulprom *et al.* 2016) that *α* > 0 can lead to substantial improvements over a purely LETKF‐based implementation and that the choice of *α* can be based on the effective sample size of the associated LETPF. However, more refined selection criteria for the parameter *α* are needed to make the hybrid LETPF‐LETKF method widely applicable.

### 5.5 Hybrid EnVar PF

Based on the localized adaptive particle filter (LAPF) described in Section 4.1.3, a hybrid particle filter‐based ensemble variational data assimilation system (PfVar) can also be constructed. The idea is to replace the LETKF‐based ensemble in an EnVar by an LAPF‐based ensemble.

We briefly discuss a practical numerical weather prediction example here. Following Buehner *et al.* (2013), the operational EnVAR system of DWD for the ICON model with 13 km global resolution and 6.5 km resolution of its two‐way nested area over Europe is using the ensemble of the global 40‐member LETKF for its dynamic covariance matrix with a ratio of 70:30 towards the classical NMC‐based covariance matrix of the three‐dimensional variational data assimilation system with 3 hr cycling interval. The LETKF ensemble is replaced by the LAPF ensemble, where the quality control of the variational high‐resolution run is used for the ensemble data assimilation system under consideration. In the current system, no recentring of the ensemble with respect to the variational mean estimator is carried out, leading to a form of weak coupling of the systems.

In a quasi‐operational set‐up (without a high‐resolution nest), the hybrid PfVAR is running stably for a period of one month. The observation minus background statistics show very promising behaviour in several case‐studies which are under investigation at DWD (Walter *et al.* 2018). In the current state of tuning, the forecast quality of the PfVAR seems comparable to the forecasts based on the LETKF‐based EnVAR. These new results studied in combination with Robert *et al.* (2018) show that today's particle filters are approaching the quality of state‐of‐the‐art operational ensemble data assimilation systems and are already becoming important tools on all scales of NWP.

### 5.6 Discussion

Hybrid particle‐ensemble Kalman filter schemes, especially when implemented adaptively, can avoid weight collapse in the particle filter part of the hybrid in any situation. The price paid is that not all information from the observations is extracted when the posterior pdf is severely non‐Gaussian, but in many situations this is not the dominant source of error. The reason why these schemes are competitive is that they do take into account some non‐Gaussianity via the particle filter, while the particle filter alone is very inefficient compared to the EnKF when the posterior is actually close to a Gaussian. So the objective is not necessarily to make the *α* as small as possible, but indeed to find an optimal *α* to ensure that the EnKF is used whenever possible. The same is true for the bridging parameter in the Adaptive Gaussian Mixture Filter.

The second‐order exact filters are hybrids of a different kind, focussing on obtaining the posterior mean and the covariance correct given the limited prior ensemble. These methods are expected to be quite competitive to the hybrid filters discussed above, and the relative performance will depend strongly on the measure used to define what is best. For instance, RMSE are expected to be better for the second‐order exact filters, while full ensemble measures like rank histograms and continuous ranked probability scores might benefit from the hybrid schemes.

One question that emerges when comparing the EnKPF and the LETPF–LETKF hybrid is which should be used first, the particle filter or the ensemble Kalman filter? Different experimental results seem to indicate that either ordering can be superior. The PF‐first methods have the advantage of a theoretical justification via a two‐step tempering interpretation in which the particle filter step makes the prior for the EnKF much more Gaussian. Applying the EnKF first will bring the particles closer to the observations, leading to better weight balance in the particle filter. At this moment it is unclear which order is best; much more research is needed.

## 6 CONCLUSIONS AND DISCUSSION

The largest issue of standard particle filters was until recently their degeneracy in high‐dimensional settings: when the number of independent observations is large and the number of particles is limited (of order 10–1,000 for geophysical applications), one particle gets weight one, and all others get weight zero.

Two developments have revived the interest in particle filters: efficient proposal densities and localization, while hybrids with EnKFs and recently transportation filters enhance confidence in the usefulness of particle filters in high‐dimensional settings. The new development is particle flow methods, whose popularity in the large machine‐learning community ensures rapid progress here, too. It is unclear at this moment how competitive these new ideas will be. It is clear that developments on particle filters have been very fast, and the first tests of both localized and hybrid particle‐EnKF filters in operational numerical weather prediction have been performed and show highly encouraging results.

This paper discussed these new developments and demonstrates that particle filters are useful in even the largest dimensional geophysical data assimilation problems and will allow us to make large steps towards fully nonlinear data assimilation. The emphasis was here on explaining and connecting existing and new ideas, including new understanding of the optimality of the optimal proposal density and equal‐weight filters.

From the presentation it has become clear that the field is too young to provide solid guidance on which method will be most fruitful for which problem. Given that most data assimilation practitioners will have an implementation of a local EnKF in some form, localized particle filters seem to be the fastest way to make progress. However, one has to keep in mind that the resampling step needs smoothing that is more complex than in an EnKF, although exciting new variants like the ETPF and LAPF allow for smooth updates in a very natural way. Furthermore, with the small ensemble sizes now practical (10–100), more than ten independent observations in a localization area may already lead to filter degeneracy, forcing us to look into methods that limit the weights from below. This is another *adhoc* procedure that limits information extraction from observations, but it is unclear how severe this issue is.

Even easier are implementations of hybrid PF‐EnKF filters, but it is still unclear what these filters target. At the moment their value lies in bringing more non‐Gaussianity into EnKFs, but at the same time ensure that an EnKF is used when that is warranted.

We discussed two main variants that try to avoid localization because of the issues discussed above: the equal‐weight particle filters and transportation particle filters. The equal‐weight variants, which avoid weight collapse by construction, do not have a complete mathematical foundation yet. We know these schemes are biased, but since they are tailored to high‐dimensional problems with small ensemble sizes, the bias error might be smaller than the Monte‐Carlo error from the small ensemble size. Transportation particle filters still have to demonstrate their full potential in geoscience applications, but initial experiments with, for example, mapping particle filters on low‐to‐moderate dimensional systems together with the way they are formulated suggest they could become mainstream competitive schemes.

All in all, huge progress has been made in particle filtering, and initial attempts to implement the schemes into full‐scale numerical weather prediction models have succeeded, with promising initial results. This shows that particle filters can no longer be ignored for high‐dimensional geoscience applications.

## APPENDIX

## LAW OF TOTAL VARIANCE

The law of total variance is an elementary theorem in statistics and probability. It can be proven as follows. First we need the Law of Total Expectation, which reads, using *E*_*A*_[*B*] as denoting the expectation of *B* under pdf *p*(*a*):

(A 1) $$\begin{array}{lll} E_{Y}[E_{X|Y}[f(X)]] & = & \int \int f(x)p(x|y)p(y)\;dx\;dy\\ & = & \int_{x}\int_{y}f(x)p(x,y)\;dy\;dx\\ & = & \int f(x)p(x)\;dx\\ & = & E_{X}[f(X)]. \end{array}$$

Using this equality on var_**X**_[**X**] leads to:

(A 2) $$\begin{array}{lll} {\mathrm{var}}_{X}[X] & = & E_{X}[X^{2}]-E_{X}^{2}[X]\\ & = & E_{Y}\left[E_{X|Y}[X^{2}]\right]-E_{Y}^{2}[E_{X|Y}[X]]\\ & = & E_{Y}\left[{\mathrm{var}}_{X|Y}[X]+E_{X|Y}^{2}[X]\right]-E_{Y}^{2}[E_{X|Y}[X]]\\ & = & E_{Y}\left[{\mathrm{var}}_{X|Y}[X]\right]+E_{Y}\left[E_{X|Y}^{2}[X]\right]-E_{Y}^{2}[E_{X|Y}[X]]\\ & = & E_{Y}\left[{\mathrm{var}}_{X|Y}[X]\right]+{\mathrm{var}}_{Y}\left[E_{X|Y}[X]\right], \end{array}$$

which proves the theorem.

## Footnotes

1 Since 20 January 2016 for the global ICON model with 40 km global ensemble resolution including a 20 km resolved two‐way nest over Europe; and since 21 March 2017 for the COSMO model with 2.8 km resolution over central Europe.
